# Cicada minimum age tree: Cryptic speciation and exponentially increasing base substitution rates in recent geologic time

**DOI:** 10.12688/f1000research.76068.5

**Published:** 2025-02-14

**Authors:** Soichi Osozawa, John Wakabayashi

**Affiliations:** 1 Tohoku University, Sendai, Japan; 2 California State University, Fresno, Fresno, USA

**Keywords:** fossil calibration, geological event calibration, exponentially increase, base substitution rate, increased biodiversity, cryptic species, ice age, C4 grasses

## Abstract

We developed a new time-calibrated phylogenetic tree incorporating primarily endemic Ryukyu Islands cicada data, along with some cryptic species, following the recent global cicada studies by Marshall *et al.* (2018), Łukasik *et al.* (2018), Simon *et al.* (2019), Price *et al.* (2019), and Hill *et al.* (2021). A total of 352 specimens were analyzed using BEAST v1.10.4 software with a relaxed clock model. Fossil calibrations dating as far back as the Triassic were adopted, largely following Johnson *et al.* (2018) and Moulds (2018), with a Quaternary geological event calibration based on Osozawa *et al.* (2012, 2021b), which was input into BEAST v1.10.4. In the COI tree, the crown age of Cicadoidea was estimated at 200.63 Ma. Tettigarctidae was found to be the oldest lineage, sister to all remaining cicadas. Derotettiginae, at 99.2 Ma, is the next oldest lineage, sister to all other monophyletic cicadas. The Tibicininae clade branched at 66.15 Ma, with the subfamilies Tettigomyiinae, Cicadettinae, and Cicadidae diverging at a crown age of 40.57 Ma. The Cicadinae clade consists of many tribe and genus-specific clades, with numerous cryptic species emerging due to vicariance and adaptive radiation. We estimated the base substitution rate as a function of age, and the results strongly indicate an exponential increase in substitution rates during recent geological time. This increase in cicada biodiversity, including the generation of cryptic species in the Ryukyu Islands and surrounding regions, may have been driven by the spread of C4 grasses and concurrent Quaternary climate changes.

## Introduction

A phylogenetic tree of worldwide cicada was recently constructed by Marshall *et al.* (2018) and Simon *et al.* (2019) applying five concatenated sequences of mitochondrial COI and COII, and nuclear ARD1, EF-1a, and 18S rRNA, and by Łukasik *et al.* (2019) applying whole mitochondrial sequences for representative species in Marshall *et al.* (2018), and family level phylogenetic relation has been clarified. Although Tettigarctinae is an old diverged lineage and Derotettiginae may be next, their worldwide phylogenetic trees were not dated trees. Price *et al.* (2019; restricted to Platypleurini) and Hill *et al.* (2021; restricted to Asian Cicadinae) built partial (not worldwide) dated trees using BEAST v.2.5 ( Bouckaert *et al.* 2014) applying COI and other sequences, but much of global cicada evolution has not been tied to absolute time.

The latest version of BEAST (Bayesian Evolutionary Analysis Sampling Trees v1. X; v1.10.4 2021; Suchard *et al.* 2018) released on 10 June 2018 has a clear and simple age calibration protocol and function, updated from BEAST v.1. 7 (v1. X ≒ v.1. 8). This calibration involves applying times of the most recent common ancestors (tMRCAs) of the ingroup species, i.e., applying the node age of a specific clade as a minimum age, in the associated software of BEAUti (Bayesian Evolutionary Analysis Utility; BEAST is the platform software). The maximum age constraint normally considered in MCMCtree (4.9e 2017; Yang 2007) was not clearly defined ( Benton & Donoghue 2007; Marshall 2008; Hill *et al.* 2021), and simply handled by ignoring the maximum age in BEAST v.1. X calibration ( Osozawa & Wakabayashi 2021; Osozawa *et al.* 2021a). We sought the oldest fossil of the corresponding node of specific clade with an assumption that the oldest fossil age was equivalent to the minimum age and equivalent to “tMRCA” in BEAST v1. X. Moulds (2018) reviewed the ages of cicada fossils. These redefined ages, ranging from 16.45 ± 0.45 Ma to 244.5 ± 2.5 Ma, were available for our fossil-based time calibrations in BEAST v1. X.

Klopfstein (2021) suggested that recent node dating approaches including Misof *et al.* (2014) and Montagna *et al.* (2019) have a credibility problem: different studies using the same molecular data and even the same sets of fossils regularly arrive at drastically different age estimates. She showed that a major reason for these differences is well known: even well-dated and firmly placed fossils can only provide a minimum age for a particular node. Therefore our fossil calibration applying solely minimum age (= tMRCA) was credible.

As shown by Osozawa *et al.* (2017a), *Platypleura* and some other endemic cicadas in Ryukyu Islands can be rigidly calibrated by a geological event calibration at 1.55 ± 0.15 Ma (Quaternary; Osozawa *et al.* 2012). As shown by Osozawa *et al.* (2021a), *Meimuna opalifera* and some other endemic cicadas on Hachijo-jima, Izu-Bonin islands, can be calibrated by a geological event calibration of emergent age at 0.24 Ma (Quaternary; Osozawa *et al.* 2021b).

**Figure 1.**
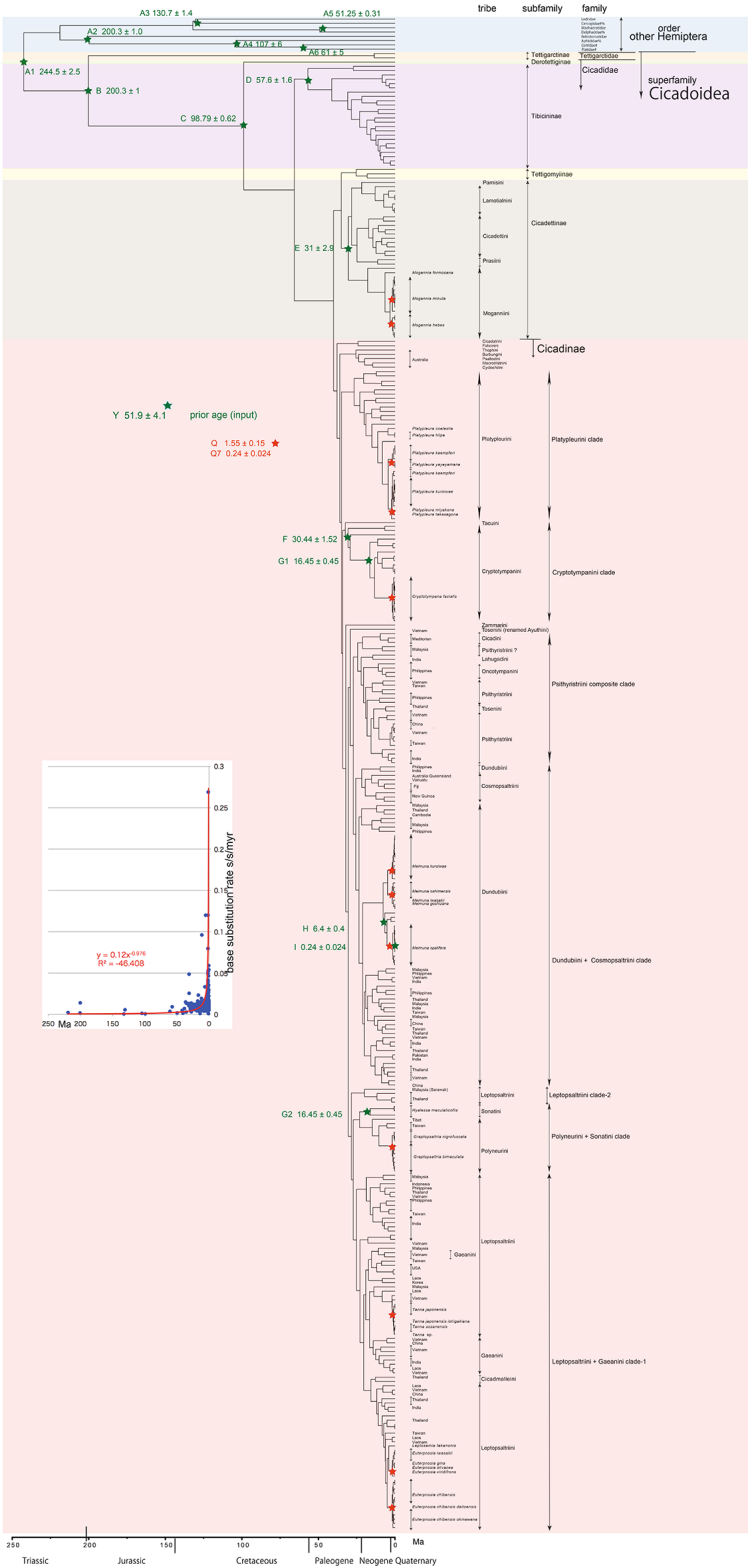
Simplified cicada timetree built by BEAST v1.X, applying a 1,534 bp in maximum COI sequence. Inserted figure: Base substitution rate (= rate median shown at each node; substitutions per site per million year; s/s/myr) vs age (= posterior age shown at each node) diagram. Red approximate curve with its formula was drawn by an Excel function, with the intersection for the curve = 0.0128 s/s/myr, the rate median shown on Tracer.

**Figure 2.**
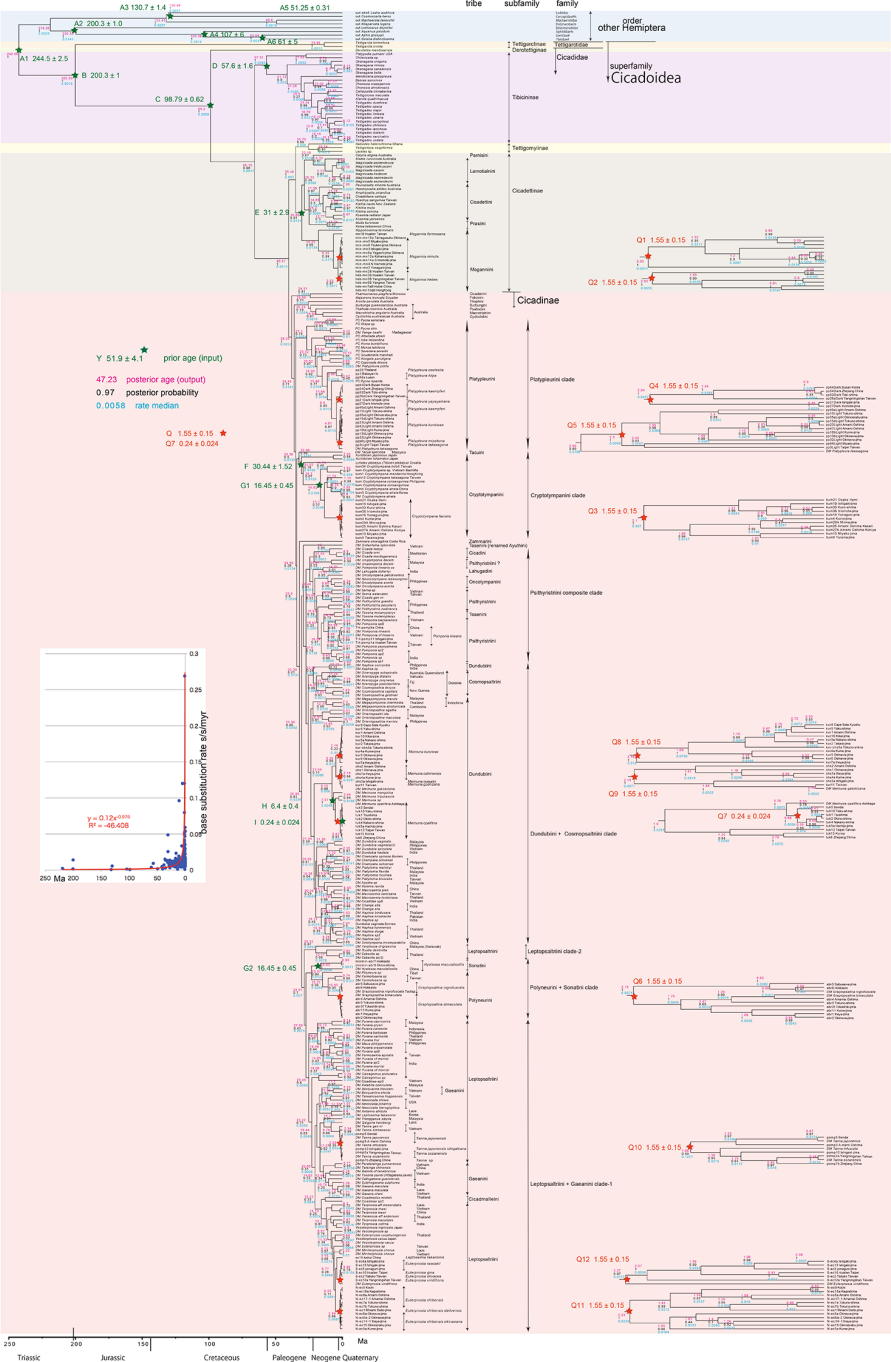
Cicada timetree built by BEAST v1.X, applying 1,534 bp COI sequence. OUTs with isolate number: our own analyzed specimens shown in Table 1, and others: from GenBank/DDJB. In outgroup Hemiptera; #: analyzed family by Johnson *et al.* (2018); % analyzed family by Misof *et al.* (2014). Inserted figure: Base substitution rate (= rate median shown at each node; substitutions per site per million year; s/s/myr) vs age (= posterior age shown at each node) diagram. Red approximate curve with its formula was drawn by Excel function, with the intersection for the curve = 0.0128 s/s/myr, the rate median shown on Tracer.

**Figure 3.**
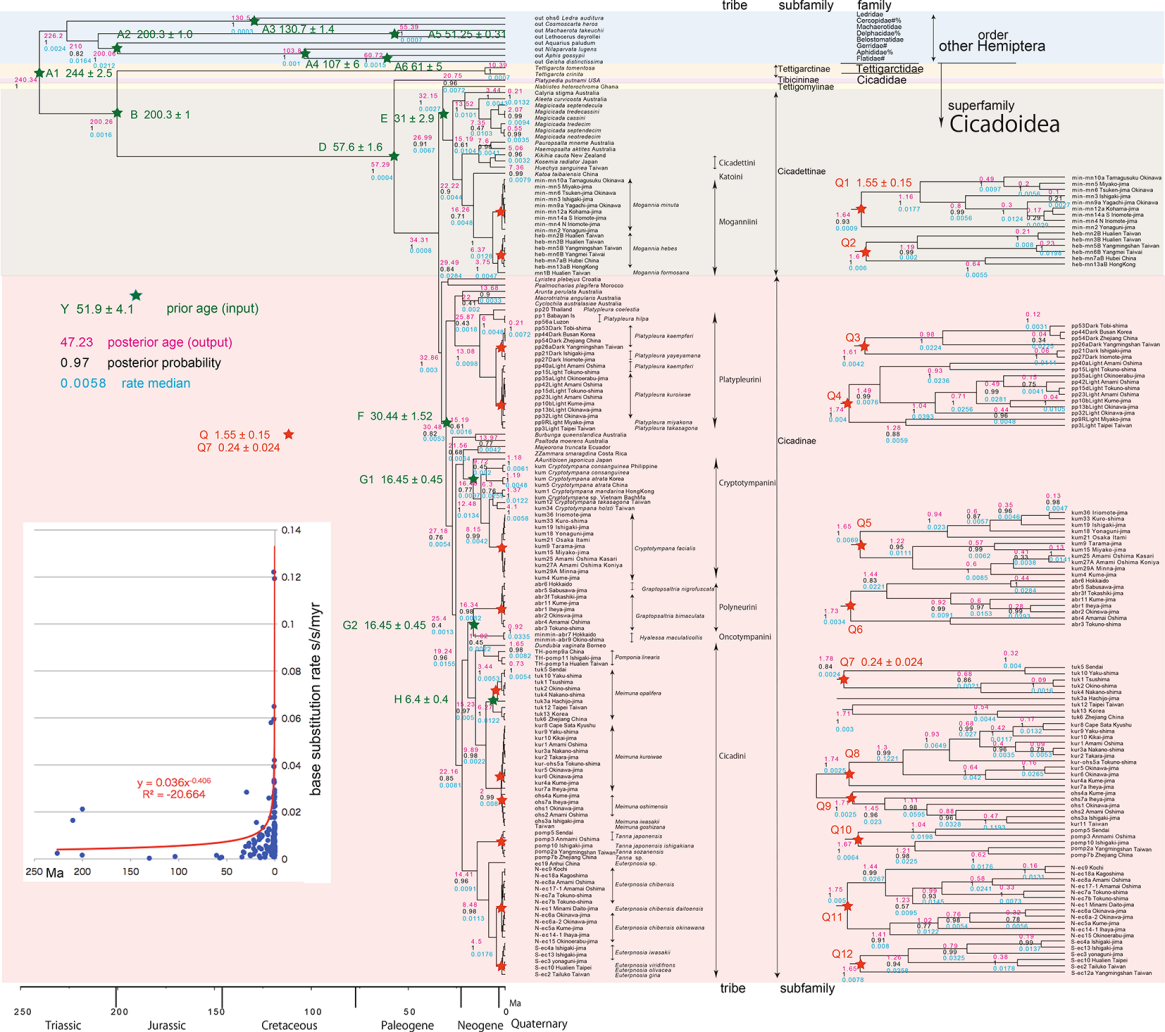
Cicada timetree built by BEAST v1.X, applying 1,534 bp COI and 874 bp 18S rRNA sequences. OUTs with isolate number: our own analyzed specimens shown in Table 1, and others: from GenBank/DDJB. In outgroup Hemiptera; #: analyzed family by Johnson *et al.* (2018); % analyzed family by Misof *et al.* (2014). Inserted figure: Base substitution rate (= rate median shown at each node; substitutions per site per million year; s/s/myr) vs age (= posterior age shown at each node) diagram. Red approximate curve with its formula was drawn by Excel function, with the intersection for the curve = 0.0114 s/s/myr, the rate median shown on Tracer. Note that this rate is a little slower than that solely of COI in Figures 1 and 2, reflecting slower rate of 18S rRNA than COI (see Osozawa *et al.* 2017a).

Through these analyses, we corroborated the classification and some rearrangement of species into four subfamilies of Tibicininae, Tettigomyiinae, Cicadettinae, and Cicadinae included in a family Cicadidae by Marshall *et al.* (2018) and Łukasik *et al.* (2018), and then estimated the splitting dates of these subfamilies, tribes (especially Cicadinae tribes after Hill *et al.* 2021), and species ( Figures 1– 3). In the BEAST analyses, we included *Derotettix*, a relict species of new subfamily Derotettiginae with the oldest lineage in family Cicadidae ( Simon *et al.* 2019), and attempted to estimate the crown age ( Figure 2). Comparison to the entire Hemipteroid insect timetree ( Johnson *et al.*, 2018) and entire insect timetree ( Misof *et al.* 2014) could be conducted as an extension of this analysis, by adding other Hemiptera species as outgroup ( Figures 1– 3).

Our primary goal was to present the precise evolutionary history of all cicadas by constructing the BEAST timetree, and also taxonomic reconsiderations for Cicadinae tribes after Hill *et al.* (2021) and for Ryukyu endemic cicadas. Another BEAST v1. X function facilitates additional evaluation of the time variability of base substitution rates. Recent dating analyses employ a relaxed clock model, which allows each branch of a phylogenetic tree to have its own evolutionary rate ( Drummond *et al.* 2012). Although the relaxed distribution can be set to lognormal in BEAUti, the rate of variability has not been documented prior to this study. The output figure of BEAST v1. X presents the base substitution rate and age at each node, and shows the acceleration of base substitution rates through the time.

## Methods

### Ethical approval

The present study did not concerned invertebrate experiments and did not involve endangered or protected species. We obtained permission of collection in the Taroko National Park, Taiwan, from the director (No. 0990012881; August 1 ~ 11, 2010), with a help of Bor-ming Jahn, and permission of collection in the Tokara islands, from the Toshima village headman, from August 29 ~ September 8, 2010. Collection in the Ryukyu islands was before the designation of National Park since 2016. No specific permission was required outside the national parks and private areas.

### Taxon sampling

Marshall *et al.* (2018), Simon *et al.* (2019), Łukasik *et al.* (2019) included comparatively few Asian cicada species in their analyses. We have previously published 70 isolate data from *Platypleura* primarily from the Japan, Ryukyu, and Taiwan islands ( Osozawa *et al.* 2017a; our aim was the vicarince acted on each island population started at 1.55 Ma and the cryptic speciation), and 21 of these data were used in the present analyses by excluding duplicated sequence data. We also collected and analyzed cicada specimens, adding isolate data from 92 specimens. Accordingly, our own data total 21 + 92 = 113 specimens ( Table 1). Note that we collected all the 35 species from Japan including the Ryukyu Islands, but excepting severely protected *Platypleura albivannata* (see Osozawa *et al.* 2017a; may be extinct without DNA sequence data) and *Meimuna boninensis* (see Osozawa *et al.* 2021a).

**Table 1.** Cicada species collected and analyzed in this paper. Specimens were mostly from the Ryukyu Islands.

isolate	country	species	accession no. COI	accession no. 18SrRNA	collection date	collected by
pp44a	South Korea:Busan	*Platypleura. kaempferi* (Fabricius, 1794)	AB897523 LC086120	LC086191	13-07-2011	Soichi Osozawa
pp53	Japan: Honshu,Tobi-shima	*Platypleura. kaempferi* (Fabricius, 1794)	LC086279 LC086066	LC086137	31-07-2014	Soichi Osozawa
pp54	China: Zhejiang	*Platypleura kaempferi* (Fabricius, 1794)	LC086280 LC086067	LC086138	13-08-2014	Akira Mishima
pp26a	Taiwan:Yangmingshan park	*Platypleura kaempferi* (Fabricius, 1794)	AB897530 LC086092	LC086163	07-06-2013	Soichi Osozawa
pp6R	Japan:Ryukyu, Ishigaki-jima	*Platypleura yayeyamana* Matsumura, 1917	AB897534 LC086072	LC086143	05-07-2010	Soichi Osozawa
pp21	Japan:Ryukyu, Ishigaki-jima	*Platypleura yayeyamana* Matsumura, 1917	AB897535 LC086085	LC086156	05-07-2010	Soichi Osozawa
pp27	Japan:Ryukyu, Iriomote-jima	*Platypleura yayeyamana* Matsumura, 1917	AB897540 LC086096	LC086167	14-06-2013	Soichi Osozawa
pp9R	Japan:Ryukyu, Miyako-jima	*Platypleura miyakona* (Matsumura, 1917)	AB897557 LC086073	LC086144	03-07-2011	Soichi Osozawa
pp3	Taiwan:Taipei, Entsu-ji	*Platypleura takasagona* Matsumura, 1917	AB897556 LC086071	LC086142	27-05-2012	Shusuke Osozawa
pp40a	Japan:Ryukyu, Amami-Oshima	*Platypleura kaempferi* Matsumura, 1917	AB897542 LC086115	LC086186	07-07-2013	Soichi Osozawa
pp15d	Japan:Ryukyu, Tokuno-shima	*Platypleura kaempferi* Matsumura, 1917	AB897548 LC086083	LC086154	02-07-2012	Soichi Osozawa
pp32	Japan:Ryukyu, Okinawa-jima	*Platypleura kaempferi* Matsumura, 1917	AB897561 LC086103	LC086174	20-06-2013	Soichi Osozawa
pp10b	Japan:Ryukyu, Kume-jima	*Platypleura kuroiwae* Matsumura, 1917	AB897563 LC086075	LC086146	23-06-2012	Soichi Osozawa
pp13b	Japan:Ryukyu, Okinawa-jima	*Platypleura kuroiwae* Matsumura, 1917	AB897562 LC086079	LC086150	22-06-2010	Soichi Osozawa
pp15d	Japan:Ryukyu, Tokuno-shima	*Platypleura kuroiwae* Matsumura, 1917	AB897565 LC086080	LC086151	02-07-2012	Soichi Osozawa
pp23	Japan:Ryukyu, Amami-Oshima	*Platypleura. kuroiwae* Matsumura, 1917	AB897566 LC086087	LC086158	23-06-2010	Soichi Osozawa
pp35a	Japan:Ryukyu, Okinoerabu-jima	*Platypleura kuroiwae* Matsumura, 1917	AB897569 LC086108	LC086179	28-06-2013	Soichi Osozawa
pp42	Japan:Ryukyu, Amami-Oshima	*Platypleura kuroiwae* Matsumura, 1917	AB897567 LC086117	LC086188	09-07-2013	Soichi Osozawa
pp20	Tailand:Chiang Rai	*Platypleura nobilis* (Germar, 1830)	AB897581 LC086133	LC086204	05-08-2008	Tetsuo Miyashita
pp1	Philippine:Babuyan island	*Platypleura hilpa* Walker, 1850	AB897582 LC086134	LC086205	05-07-2011	Kei Nishiguro
pp56a	Philippine:Luzon island, Mt. Mayon	*Platypleura hilpa* Walker, 1850	(AB897582) LC086135	LC086206	05-04-2014	Kei Nishiguro
kum25	Japan:Ryukyu, Amami-Oshima	*Cryptotympana facialis* (Walker, 1858)	LC466803	LC466820	05-07-2018	Kenichi Kanai
kum27A	Japan:Ryukyu, Amami-Oshima	*Cryptotympana facialis* (Walker, 1858)	LC466804	LC466821	06-07-2018	Kenichi Kanai
kum4	Japan:Ryukyu, Kume-jima	*Cryptotympana facialis* (Walker, 1858)	LC466805	LC466822	05-07-2014	Fumiyasu Sato
kum29A	Japan:Ryukyu, Okinawa-jima, Minna-jima	*Cryptotympana facialis* (Walker, 1858)	LC466806	LC466823	28-06-2018	Satoru Nitta
kum15	Japan:Ryukyu, Miyako-jima	*Cryptotympana facialis* (Walker, 1858)	LC466807	LC466824	04-07-2011	Soichi Osozawa
kum9	Japan:Ryukyu, Tarama-jima	*Cryptotympana facialis* (Walker, 1858)	LC466808	LC466825	14-06-2016	Soichi Osozawa
kum19	Japan:Ryukyu, Ishigaki-jima	*Cryptotympana facialis* (Walker, 1858)	LC466809	LC466826	26-06-2017	Hiroshi Irino
kum33	Japan:Ryukyu, Kuro-shima	*Cryptotympana facialis* (Walker, 1858)	LC466810	LC466827	18-07-2018	Soichi Osozawa
kum36A	Japan:Ryukyu, Iriomote-jima	*Cryptotympana facialis* (Walker, 1858)	LC466811	LC466828	07-09-2018	Hiroshi Irino
kum18	Japan:Ryukyu, Yonaguni-jima,	*Cryptotympana facialis* (Walker, 1858)	LC466812	LC466829	10-07-2019	Minoru Saijo
kum21	Japan: Honshu,Itami	*Cryptotympana facialis* (Walker, 1858)	LC466813	LC466830	21-07-2018	Soichi Osozawa
kum34	Taiwan:Wulai	*Cryptotympana holsti* Distant, 1904	LC466814	LC466831	04-07-2018	Soichi Osozawa
kum1	China: HongKong	*Cryptotympana mandarina* Distant, 1891	LC466815	LC466832	29-05-2013	Soichi Osozawa
kum12	Taiwan:Taipei Zoo	*Cryptotympana takasagona* Kato, 1925	LC466816	LC466833	28-08-2016	Soichi Osozawa
kum3	South Korea:Busan	*Cryptotympana atrata* (Fabricius, 1775)	LC466817	LC466834	12-07-2013	Soichi Osozawa
kum5	China: Zhejiang	*Cryptotympana atrata* (Fabricius, 1775)	LC466818	LC466835	13-08-2013	Akira Mishima
abr5	Japan: Honshu,Sabusawa-shima	*Graptopsaltria nigrofuscata* (Motschulsky, 1866)	LC466819	LC466836	30-07-2013	Soichi Osozawa
N-ec1	Japan:Ryukyu,Minami Daito-jima	*Euterpnosia chibensis daitoensis* Matsumura, 1917	LC508809	LC508884	05-04-2012	Ryosuke Sadaki
S-ec2	Taiwan:Tailuko	*Euterpnosia olivacea* Kato, 1927	LC508810	LC508885	2010,8.8	Soichi Osozawa
S-ec3	Japan:Ryukyu,Yonaguni-jima	*Euterpnosia iwasakii* (Matsumura, 1913)	LC508811	LC508886	29-06-2011	Soichi Osozawa
S-ec4a	Japan:Ryukyu,Ishigaki-jima	*Euterpnosia iwasakii* (Matsumura, 1913)	LC508812	LC508887	25-06-2011	Soichi Osozawa
N-ec5a	Japan:Ryukyu,Kume-jima	*Euterpnosia chibensis okinawana* Ishihara, 1968	LC508813	LC508888	23-06-2012	Soichi Osozawa
N-ec6a	Japan:Ryukyu,Okinawa-jima	*Euterpnosia chibensis okinawana* Ishihara, 1968	LC508814	LC508889	25-06-2010	Soichi Osozawa
N-ec6a-2	Japan:Ryukyu,Okinawa-jima	*Euterpnosia chibensis okinawana* Ishihara, 1968	LC508815	LC508890	25-06-2010	Soichi Osozawa
N-ec7a	Japan:Ryukyu,Tokuno-shima	*Euterpnosia chibensis* Matsumura, 1917	LC508816	LC508891	02-07-2012	Soichi Osozawa
N-ec7b	Japan:Ryukyu,Tokuno-shima	*Euterpnosia chibensis* Matsumura, 1917	LC508817	LC508892	02-07-2012	Soichi Osozawa
N-ec8a	Japan:Ryukyu,Amami Oshima	*Euterpnosia chibensis* Matsumura, 1917	LC508818	LC508893	28-06-2010	Soichi Osozawa
N-ec9	Japan:Honshu,Kochi	*Euterpnosia chibensis* Matsumura, 1917	LC508819	LC508894	01-08-2011	Soichi Osozawa
S-ec10	Taiwan:Hualien	*Euterpnosia viridifrons* Matsumura, 1917	LC508820	LC508895	04-06-2013	Soichi Osozawa
S-ec12a	Taiwan:Yanhmungshan	*Euterpnosia gina* Kato, 1931	LC508821	LC508896	07-06-2013	Soichi Osozawa
S-ec13	Japan:Ryukyu,Ishigaki-jima	*Euterpnosia iwasakii* (Matsumura, 1913)	LC508822	LC508897	15-06-2013	Soichi Osozawa
N-ec14-1	Japan:Ryukyu,Ihaya-jima	*Euterpnosia chibensis okinawana* Ishihara, 1968	LC508823	LC508898	21-06-2013	Soichi Osozawa
N-ec15	Japan:Ryukyu,Okinoerabu-jima	*Euterpnosia chibensis okinawana* Ishihara, 1968	LC508824	LC508899	28-06-2013	Soichi Osozawa
N-ec17-1	Japan:Ryukyu,Amamai Oshima	*Euterpnosia chibensis* Matsumura, 1917	LC508825	LC508900	09-07-2013	Soichi Osozawa
N-ec18a	Japan:Kyushu,Kagoshima	*Euterpnosia chibensis* Matsumura, 1917	LC508826	LC508901	03-08-2013	Heruhiko Fukuda et al
ec19	China:Anhui	*Euterpnosia* sp.	LC508827	LC508902	10-06-2013	Akira Mishima
min-mn2	Japan:Ryukyu,Yonaguni-jima	*Mogannia minuta* (Matsumura, 1907)	LC508828	LC508903	29-06-2011	Soichi Osozawa
min-mn3	Japan:Ryukyu,Ishigaki-jima	*Mogannia minuta* (Matsumura, 1907)	LC508829	LC508904	01-05-2010	Soichi Osozawa
min-mn4	Japan:Ryukyu,N Iriomote-jima	*Mogannia minuta* (Matsumura, 1907)	LC508830	LC508905	30-04-2010	Soichi Osozawa
min-mn5	Japan:Ryukyu,Miyako-jima	*Mogannia minuta* (Matsumura, 1907)	LC508831	LC508906	25-04-2011	Soichi Osozawa
min-mn6	Japan:Ryukyu, Okinawa-jima, Tsuken-jima	*Mogannia minuta* (Matsumura, 1907)	LC508832	LC508907	06-06-2012	Satoru Nitta
min-mn9a	Japan:Ryukyu, Okinawa-jima, Yagachi-jima	*Mogannia minuta* (Matsumura, 1907)	LC508833	LC508908	11-05-2014	Atsuko Nitta
min-mn10a	Japan:Ryukyu,Okinawa-jima, Tamagusuku	*Mogannia minuta* (Matsumura, 1907)	LC508834	LC508909	30-05-2014	Ysushi Watanabe
min-mn12a	Japan:Ryukyu,Kohama-jima	*Mogannia minuta* (Matsumura, 1907)	LC508835	LC508910	06-07-2014	Soichi Osozawa
min-mn14a	Japan:Ryukyu,S Iriomote-jima	*Mogannia minuta* (Matsumura, 1907)	LC508836	LC508911	18-06-2016	Soichi Osozawa
mn1B	Taiwan:Hualien	*Mogannia formosana* Matsumura, 1907	LC508837	LC508912	02-06-2013	Soichi Osozawa
heb-mn2B	Taiwan:Hualien	*Mogannia hebes* Walker, 1858	LC508838	LC508913	02-06-2013	Soichi Osozawa
heb-mn3B	Taiwan:Hualien	*Mogannia hebes* Walker, 1858	LC508839	LC508914	03-06-2013	Soichi Osozawa
heb-mn5B	Taiwan:Yangmingshan	*Mogannia hebes* Walker, 1858	LC508840	LC508915	07-06-2013	Soichi Osozawa
heb-mn6B	Taiwan:Yangmei	*Mogannia hebes* Walker, 1858	LC508841	LC508916	08-06-2013	Soichi Osozawa
heb-mn7aB	China:HongKong	*Mogannia hebes* Walker, 1858	LC508842	LC508917	30-05-2013	Akira Mishima
heb-mn13aB	China:Hubei	*Mogannia hebes* Walker, 1858	LC508843	LC508918	18-06-2014	Soichi Osozawa
tuk1	Japan:Kyushu,Tsushima	*Meimuna opalifera* (Walker, 1850)	LC508844	LC508919	18-07-2013	Soichi Osozawa
tuk2	Japan:Honshu,Okino-shima	*Meimuna opalifera* (Walker, 1850)	LC508845	LC508920	28-08-2013	Soichi Osozawa
tuk3a	Japan:Izu,Hachijo-jima	*Meimuna opalifera* (Walker, 1850)	LC508846	LC508921	16-07-2014	Soichi Osozawa
tuk4	Japan:Tokara,Nakano-shima	*Meimuna opalifera* (Walker, 1850)	LC508847	LC508922	31-08-2010	Soichi Osozawa
tuk5	Japan:Honshu,Sendai	*Meimuna opalifera* (Walker, 1850)	LC508848	LC508923	06-09-2014	Soichi Osozawa
tuk6	China:Zhejiang	*Meimuna opalifera* (Walker, 1850)	LC508849	LC508924	13-08-2014	Soichi Osozawa
tuk10	Japan:Kyushu,Yaku-shima	*Meimuna opalifera* (Walker, 1850)	LC508850	LC508925	11-10-2017	Haruo Fukuda
tuk12	Taiwan:Taipei	*Meimuna opalifera* (Walker, 1850)	LC508851	LC508926	06-11-2017	Soichi Osozawa
ohs1	Japan:Ryukyu,Okinawa-jima	*Meimuna oshimensis* (Matsumura, 1905)	LC508852	LC508927	23-06-2010	Soichi Osozawa
ohs2	Japan:Ryukyu,Amami Oshima	*Meimuna oshimensis* (Matsumura, 1905)	LC508853	LC508928	09-09-2010	Soichi Osozawa
ohs3a	Japan:Ryukyu,Ishigaki-jima	*Meimuna iwasakii* (Matsumura, 1913)	LC508854	LC508929	05-09-2014	Tadafumi Nakada
ohs4a	Japan:Ryukyu,Kume-jima	*Meimuna oshimensis* (Matsumura, 1905)	LC508855	LC508930	15-08-2014	Soichi Osozawa
ohs7a	Japan:Ryukyu,Iheya-jima	*Meimuna oshimensis* (Matsumura, 1905)	LC508856	LC508930	18-09-2015	Soichi Osozawa
kur1	Japan:Ryukyu,Amami Oshima	*Meimuna kuroiwae* (Matsumura, 1917)	LC508857	LC508931	09-09-2010	Soichi Osozawa
kur2	Japan:Tokara,Takara-jima	*Meimuna kuroiwae* (Matsumura, 1917)	LC508858	LC508932	03-09-2010	Soichi Osozawa
kur3a	Japan:Tokara,Nakano-shima	*Meimuna kuroiwae* (Matsumura, 1917)	LC508859	LC508933	29-08-2010	Soichi Osozawa
kur4a	Japan:Ryukyu,Kume-jima	*Meimuna kuroiwae* (Matsumura, 1917)	LC508860	LC508934	15-08-2014	Soichi Osozawa
kur5	Japan:Ryukyu,Okinawa-jima	*Meimuna kuroiwae* (Matsumura, 1917)	LC508861	LC508935	17-09-2015	Soichi Osozawa
kur6	Japan:Ryukyu,Okinawa-jima	*Meimuna kuroiwae* (Matsumura, 1917)	LC508862	LC508936	17-09-2015	Soichi Osozawa
kur7a	Japan:Ryukyu,Iheya-jima	*Meimuna kuroiwae* (Matsumura, 1917)	LC508863	LC508937	18-09-2015	Soichi Osozawa
kur8	Japan:Kyushu,Kagoshima,Cape Sata	*Meimuna kuroiwae* (Matsumura, 1917)	LC508864	LC508938	04-10-2017	Nobuharu Kumagai
kur9	Japan:Kyushu,Yaku-shima	*Meimuna kuroiwae* (Matsumura, 1917)	LC508865	LC508939	10-10-2017	Haruo Fukuda
kur10	Japan:Ryukyu,Kikai-jima	*Meimuna kuroiwae* (Matsumura, 1917)	LC508866	LC508940	10-11-2017	Nobuharu Kumagai
TH-pomp1a	Taiwan:Hualien	*Pomponia linearis* Walker, 1850	LC508867	LC508941	04-06-2013	Soichi Osozawa
TH-pomp11	Japan:Ryukyu,Ishigaki-jima	*Pomponia linearis* Walker, 1850	LC508868	LC508942	21-08-2014	Tadafumi Nakada
TH-pomp9a	China:Zhejiang	*Pomponia linearis* Walker, 1850	LC508869	LC508943	25-07-2014	Akira Mishima
pomp3	Japan:Ryukyu,Amami Oshima	*Tanna japonensis* Distant, 1892	LC508870	LC508944	07-07-2013	Soichi Osozawa
pomp5	Japan:Honshu,Sendai	*Tanna japonensis* Distant, 1892	LC508871	LC508945	21-07-2014	Soichi Osozawa
pomp7b	China: Zhejiang	*Tanna* sp.	LC508872	LC508946	13-08-2014	Akira Mishima
pomp10	Japan:Ryukyu, Ishigaki-jima	*Tanna japonensis ishigakiana* (Kato, 1960)	LC508873	LC508947	21-08-2014	Tadafumi Nakada
pomp2a	Taiwan:Yangmingshan	*Tanna sozanensis* Kato, 1926	LC508874	LC508948	07-06-2013	Soichi Osozawa
abr1	Japan:Ryukyu, Iheya-jima	*Graptopsaltria bimaculata* Kato, 1925	LC508875	LC508949	21-06-2013	Soichi Osozawa
abr2	Japan:Ryukyu, Okinawa-jima	*Graptopsaltria bimaculata* Kato, 1925	LC508876	LC508950	22-06-2010	Soichi Osozawa
abr3f	Japan:Ryukyu, Tokashiki-jima	*Graptopsaltria bimaculata* Kato, 1925	LC508877	LC508951	24-06-2013	Soichi Osozawa
abr11	Japan:Ryukyu, Kume-jima	*Graptopsaltria bimaculata* Kato, 1925	LC508878	LC508952	22-06-2014	Fumiyasu Sato
abr3	Japan:Ryukyu, Tokuno-shima	*Graptopsaltria bimaculata* Kato, 1925	LC508879	LC508953	05-07-2013	Soichi Osozawa
abr4	Japan:Ryukyu, Amami-Oshima	*Graptopsaltria bimaculata* Kato, 1925	LC508880	LC508954	08-07-2013	Soichi Osozawa
abr6	Japan:Hokkaido	*Graptopsaltria nigrofuscata* (Motschulsky, 1866)	LC508881	LC508955	16-08-2013	Shusuke Osozawa
minmin-abr7	Japan:Hokkaido	*Hyalessa maculaticollis* (Motschulsky, 1866)	LC508882	LC508956	16-08-2013	Shusuke Osozawa
minmin-abr9	Japan:Honshu,Okino-shima	*Hyalessa maculaticollis* (Motschulsky, 1866)	LC508883	LC508957	28-08-2013	Soichi Osozawa

We incorporated representative sequence data from the GenBank/DDJB. This is because Tettigarctinae, Derotettiginae, Tibicininae, and Tettigomyiinae are not known from East Asia, and Cicadettinae has only two species of *Kosemia* in the Japan main islands. Thus to extend our analyses beyond East Asia, the Marshall *et al.* (2018) and Łukasik *et al.* (2019) data were essential for us. We combined our data from 113 East Asian specimens with data from 75 specimens from the studies of Marshall *et al.* (2018; including 20 East Asian specimens), and Łukasik *et al.* (2018; including 27 East Asia specimens). In addition, we incorporated data of 15 Platypleurini (other than *Platypleura*) from Price *et al.* (2019), and data of 149 Asian Cicadinae from recently published Hill *et al.* (2021). Accordingly we analyzed sequence data from 113 + 75 + 15 + 149 = 352 specimens.

*Platypleura* cicada ( Osozawa *et al.* 2017a) experienced vicariance triggered by the 1.55 ± 0.15 Ma isolation of the Ryukyu, Japan, and Taiwan islands from Chinese continent ( Osozawa *et al.* 2012), and we collected specimens from each island population for each *Platypleura* species. Similarly, we collected cicada specimens for the present analyses from each island population of *Mogannia* (Cicadettinae), and *Cryptotympana*, *Graptopsaltria*, *Hyalessa*, *Pomponia*, *Meimuna*, *Tanna*, and *Euterpnosia* (Cicadinae). *Hyalessa maculaticollis* was known to be affected by vicariance within China and Japan ( Liu *et al.* 2018). Our 113 East Asian specimens consist primarily of these endemic and cryptic species inhabiting Japan, Taiwan, and the Ryukyu islands.

### DNA sequence

COI and 18S rRNA sequence data from our collected 113 isolates, including *Platypleura* in Osozawa *et al.* (2017a), are shown in Table 1. Primers used, amplifications, and sequencing are given in Osozawa *et al.* (2017a). These sequence data were aligned by ClustalW in MEGA 5 ( Tamura *et al.* 2011). The COI sequence data comprise 1,534 bp, and the 18S rRNA sequence 874 bp, with high enough resolution to construct a phylogenetic tree, as we showed previous analyses of *Platypleura* ( Osozawa *et al.* 2017a). We did not analyze calmodulin in Osozawa *et al.* (2017a), because the resolution was insufficient. The COI data in Marshall *et al.* (2018) comprised 1,485 pb, comparable to ours. The COI data in Price *et al.* (2019) comprised 940 bp, and Hill *et al.* (2021) comprised 648 bp, comparable to ours, so we incorporated these data into our present analyses. Nuclear 18S rRNA shows less variation with much slower base substitution rate compared to mitochondrial COI ( Osozawa *et al.* 2017a; COI: 0.0270 substitutions/site/myr, 18S rRNA: 0.000492 s/s/myr; strict clock model; solely calibrated by 1.55 ± 0.15 Ma following Osozawa *et al.* 2012). The tree topology was unaffected by 18S rRNA ( Osozawa *et al.* 2017a), but 18S rRNA was included in the analyses in this paper.

We used COI and 18S rRNA sequence data, from 352 total specimens (239 from GenBank/DDBJ + 113 of our own) for the COI timetree in Figures 1 and 2, and 155 total specimens (42 from GenBank/DDBJ + 113 of our own) for the COI +18S rRNA timetree in Figure 3. The COI and 18S rRNA data in table 1 in Marshall *et al.* (2018) contain missing and incomparable data, so some of their GenBank/DDBJ data were not applicable for our analyses. Whole mitochondrial sequence data by Łukasik *et al.* (2019) are included in our analyses as corresponding COI regions. Within COI sequence data in Marshall *et al.* (2018), 21 data for Cicadettinae and 14 data for Cicadinae were incorporated into our analyses. 18S rRNA sequence in Marshall *et al.* (2018) was used for only *Nablistes heterochroma* (Tettigomyiinae) and *Platypedia putnami* (Tibicininae). Only the COI sequence data in Price *et al.* (2019) for Platypleurini and in Hill *et al.* (2021) for Asian Cicadinae were applied to our study.

North American Cryptotympanini were analyzed by Hill *et al.* (2015), applying 1,467 bp of COI and 783 bp of nuclear EF-1a with sufficient resolution. Cicadettini, primarily from Australia, was analyzed by Marshall *et al.* (2016), applying 1,492 bp of COI and 1,047 bp of nuclear EF-1a also with sufficient resolution. Some of these COI sequence data were included in our analyses.

For our initial analysis, we constructed a minimum age tree solely applying COI sequence data ( Figures 1 and 2; 352 specimens) that covers Tettigomyiinae and Tibicininae species. Following this analysis, we constructed a minimum age tree by applying both COI and 18S rRNA sequences ( Figure 3; 155 specimens, i.e., 352 − 155 = 197 specimens lack 18S rRNA sequences). These analyses showed that topology and ages associated with the analyses were not impacted by inclusion or exclusion of 18S rRNA sequence data.

### Why was BEAST2 not used?

Regarding BEAST2 (= *BEAST2, StarBEAST2), our approach diverged from previous studies such as Osozawa *et al.* (2016), Price *et al.* (2019), and Hill *et al.* (2021), who utilized BEAST v2.5 ( Bouckaert *et al.*, 2014). Instead, we opted for BEAST v1.8 and subsequently v1. X. While the calibration function in BEAUti of BEAST v2.5 bears similarities to BEAST v1. X, there are notable differences. In BEAST v2.5, the “Partition” tab only permits the input of individual sequence data. Consequently, if the sequence data are not concatenated, separate BEAST runs must be conducted for each set of applied sequence data (e.g., mitochondrial COI and nuclear 28S rRNA), as demonstrated by Osozawa *et al.* (2016). The resulting tree files from these runs must then be combined into a single file using LogCombiner. However, when merging these tree files, the branches in the resultant tree become folded, reflecting the incongruent topology arising from different sequence data sources, such as mitochondrial COI and nuclear 28S rRNA. To mitigate this issue, Osozawa *et al.* (2016) employed DensiTree to obscure the foldings. Consequently, we discourage the usage of BEAST v2.5 due to the inconvenience and potential confusion caused by folded branches in the combined tree.

In the case of BEAST v2.6, which was released in May 2019, and BEAST v2.7, released in 2023 ( Bouckaert *et al.*, 2019), significant changes were made to the protocols. A tutorial for these versions can be found in https://taming-the-beast.org/tutorials/starbeast2-tutorial. Notably, the inclusion of cladistic data alongside molecular data became possible with the implementation of total-evidence dating ( Zhang *et al.*, 2016). For extinct species, tip dating is set at their youngest fossil age, while for extant species, it is set at zero age. However, the fossil age is often poorly constrained, with minimum and maximum age ranges typically used. In BEAST v2.6 and v2.7, the calibration and node dating function that was implemented in BEAST v2.5 was abandoned, and node dating for extant species is solely based on applying and assuming the base substitution rate.

In the context of BEAST v2.6 and v2.7, it is important to clarify that the term “tip” does not refer to terminal nodes for extant species. Instead, it refers to the tip node representing extinct fossil species from ancient times (c.f., https://beast.community/first_tutorial). The tip date for fossil species is inferred from the fossil age, and it is worth noting that the age assigned is not necessarily the minimum age for the oldest fossil, but rather the youngest fossil, which is often poorly constrained. Additionally, it is crucial to ensure that these fossil species are indeed extinct, and determining their relative placement in relation to the lineage of extant species can be problematic, as it involves the concept of ghost lineage. It is important to understand that tip dating does not contribute to the quality of node dating.

### Phylogenetic analyses by BEAST v1. X

A Bayesian inference (BI) tree ( Figures 1– 3) was constructed using the software BEAST v1. X, running BEAUti, BEAST, TreeAnnotator, and FigTree, in ascending order. Before operating the BEAST software, the BEAGLE Library must be downloaded. Tracer v.1.6 was applied for checking the calculation status and estimating the median base substitution rate.

For graphic explanation of the operation of this software, see Osozawa (2021a; BEAST v1.X tutorial, in a case of four cicada genera) at: dx.doi.org/10.17504/protocols.io.bq6mmzc6.

In BEAUti, the following software settings were used (Appling Appendix BEAUti file, readers may run the platform software BEAST and check the protocol and reliability).

Partitions: Loading fasta files was by using the Import Data or plus button. Partitions defined by the COI and 18S rRNA gene sequences appeared in the Partition box (For Figure 3; COI file only for Figures 1 and 2). Note that COI and 18S rRNA partitions automatically appear in Partitions without employing PartitionFinder, and the partitioning is performed simply by applying each COI and 18S rRNA sequence, instead of the concatenating of genes by SeaView ( Gouy *et al.* 2010) as done by Price *et al.* (2019) and Hill *et al.* (2021). Additional partitioning by PartitionFinder 2 ( Lanfear *et al.* 2016) in MCMCTree and BEAST2 analyses is not required in the present BEAST1 analyses.

Taxa: Loading of taxa as ingroup was by using the plus button. The left screen: Taxon Set (monophyletic boxes were checked for all, and stem box were checked in case by case; see Table 2), and the right screen: Included monophyletic Taxa (= specific clade) and the resting Excluded Taxa in the central screen. As input in Figures 1– 3, calibration dates were set in Priors bellow.

**Table 2.** Hemiptera, mostly Cicadomorpha calibrations. These are primarily fossil calibrations but include geological event calibrations. See main text and Figures 1– 3.

Calibration point	Fossil	Subfamily	Family	Infraorder-suborder	Order	Ingroup clade	Johnson *et al.* (2018) X Moulds (2018) Y	Formation	System	Stage	tMRCA (Ma)	Method	Paleontological reference	Geological reference
A1	† *Vosegus triassicus*		Aphidoidea others	Aphidomorpha	Hemiptera	Aphidoidea others (stem)	X	Bundsandstein	Triassic	Anisian	244.5 ± 2.5	correlation	Szwedo & Nel (2011)	established
A2	† *Odrowazicoris polonicus*		Belostomatidae	Nepomorpha	Hemiptera	*Lethocerus deyrollei* (stem)		Zagaje Formation	Jurassic	Hettangian	200.3 ± 1.0	lacking	Popov (1996)	lacking
A3	†		Ledridae Cercopidae	Cicadomorpha	Hemiptera	*Ledra auditura* (stem)		Jehol Biota	Cretaceous	Hauterivian	130.7 ± 1.4	Ar-Ar dating	Zhang (1997) Hong (1982)	He *et al.* (2006)
A4	† *Cretogerris albianus*		Gerridae	Gerromorpha	Hemiptera	*Aphis gossypii* (strem)	X	French amber	Cretaceous	Albian	107 ± 6	lacking	Perrichot *et al.* (2005)	lacking
A5	†		Delphacidae	Fulgoromorpha	Hemiptera	*Nilaparvata lugens* (stem)		Green River	Paleogene	Eocene Yepresian	51.25 ± 0.31	Ar-Ar dating	Grande (1980)	Smith *et al.* (2003)
A6	† *Ormenis devincta*		Flatidae	Fulgoromorpha	Hemiptera	*Geisha distinctissima* (stem)	X	Maíz Gordo Formation	Paleogene	Paleocene	61 ± 5	lacking	Petrulevičius (2011)	lacking
B	† *'Liassocicada' ignota*	Tettigarctinae	Tettigarctidae	Cicadomorpha	Hemiptera	*Epiophlebia superstes* (stem)	Y	Dorset	Jurassic	Hettangian	203.1± 1.0	correlation	Whalley (1985)	established
C	† *Burmacicada protera*	Derotettiginae	Cicadidae	Cicadomorpha	Hemiptera	*Derotettix mendosensis* (stem)	Y	Burmese amber	Cretaceous	Cenomanian	98.79 ± 0.62	U-Pb dating	Poinar & Kritsky (2011)	Shi *et al.* (2012)
C (not applied)	Amaranthaceae	Derotettiginae	Cicadidae	Cicadomorpha	Hemiptera	*Derotettix mendosensis* (stem)		Koluel-Kaike Formation	Paleogene	Eocene	49.512 ± 0.019	Ar-Ar dating	Zucol *et al.* (2018)	Woodburne *et al.* (2014)
D	† *Davispia bearcreekensis*	Tibicininae	Cicadidae	Cicadomorpha	Hemiptera	Tibicininae	Y	Fort Union Formation	Paleogene	Paleocene	57.6 ± 1.6	correlation	Cooper (1941)	Flores & Bader (1999)
D (not applied)	† *Platypedia primigenia*	Tibicininae	Cicadidae	Cicadomorpha	Hemiptera	Tibicininae	Y	Florissant Formation	Paleogene	Oligocene	35.15 ± 1.65	Ar-Ar dating	Mcintosh *et al.* (1992)	Mcintosh *et al.* (1992)
D (not applied)	† *Hadoa grandiose*	Tibicininae	Cicadidae	Cicadomorpha	Hemiptera	Tibicininae	Y	Florissant Formation	Paleogene	Oligocene	35.15 ± 1.65	Ar-Ar dating	Mcintosh *et al.* (1992)	Mcintosh *et al.* (1992)
E	† *Paracicadetta oligocenica*	Cicadettinae	Cicadidae	Cicadomorpha	Hemiptera	Cicadettinae	Y	Créste	Neogene	Oligocene Rupelian	28.465 ± 5.435	correlation	Boulard & Nel (1990)	Ducreux *et al.* (1985)
F	† *Lyristes* sp.	Cicadinae	Cicadidae	Cicadomorpha	Hemiptera	*Lyristes plebejus* (stem)	Y	Seifhennersdorf	Jirassic	Tithonian	30.44 ± 1.52	K-Ar dating	Tietz *et al.* (1998)	Walther & Kvacek (2007)
G1	† *Cryptotympana incasa*	Cicadinae	Cicadidae	Cicadomorpha	Hemiptera	*Cryptotympana* spp.	Y	Shanwang	Neogene	Miocene Langhian	16.45 ± 0.45	correlation	Zhang *et al.* (1994)	Roček *et al.* (2011)
G2	† *Hyalessa lapidescens*	Cicadinae	Cicadidae	Cicadomorpha	Hemiptera	*Hyalessa maculaticollis*	Y	Shanwang	Neogene	Miocene Langhian	16.45 ± 0.45	correlation	Zhang (1989)	Roček *et al.* (2011)
H	† *Meimuna protopalifera*	Cicadinae	Cicadidae	Cicadomorpha	Hemiptera	*Meimuna* spp.	Y	Zhirkindek	Neogene	Miocene Messinian	6.4 ± 0.4	fission track	Fujiyama (1969)	Fujiwara et al. (2008)
Q7	geological event	Cicadinae	Cicadidae	Cicadomorpha	Hemiptera	*Meimuna opalifera*		Hachijo-jima	Quaternary	Pleistocene Chibanian	0.024 ± 0.0024	U-Pb dating	Osozawa *et al.* (2021b)	Osozawa et al. (2021b)
Q1-6, Q8-12	geological event	Cicadinae	Cicadidae	Cicadomorpha	Hemiptera	*Meimuna opalifera* and others		Ryukyu	Quaternary	Pleistocene Calabrian	1.55 ± 0.15	biostratigraphy	Osozawa *et al.* (2012)	Osozawa et al. (2012)

Tips and Traits: Default.

Sites: Substitution Model: HKY (Hasegawa, Kishino and Yano) model, Base frequencies: Empirical, Site Heterogenety Model: Gamma, Number of Gamma Categories: 4, Partition into codon positions: Off. The GTR model generates similar topology.

Clocks: Clock Type: Uncorrected relaxed clock, Relaxed Distribution: Lognormal. Uncorrelated relaxed clocks allow each branch of a phylogenetic tree to have its own evolutionary rate under log-normal distribution, and the node rate is the rate median of three branches (Drummond *et al.* 2006).

Trees: Tree Prior: Speciation: Yule Process.

Priors: tMRCA (time of MRCA) was input from the calibration point date as Prior Distribution: Normal, and as the Mean and Standard deviation. See bellow for Priors as detailed setting of age calibration.

Operators: Default.

MCMC: Length of chain: 10,000,000.

Running BEAST was done by incorporating xml input file made by BEAUti. The consequent tree was drawn by FigTree v1.4.2, for that, the tree files were input into TreeAnnotator. The 95% highest posterior density for confidence intervals of ages can be output in FigTree, but not shown in Figures 1– 3 to avoid visual complexity. In FigTree, posterior probability (“posterior”), posterior age (“Node ages”), and “rate median” (not constant) can be output, and these are shown at each node in Figures 2 and 3. This rate related function was not used in any previous paper, and we found in this paper variable base substitution rates the time as suggested by the relaxed clock model of BEAST ( Drummond *et al.* 2012). Consequently, we made base substitution rate (“rate median” shown at each node in FigTree) vs age (“Node age” shown at each node in FigTree) diagram ( Figures 1– 3 inset) using a function of Excel.

**Figure 4.**
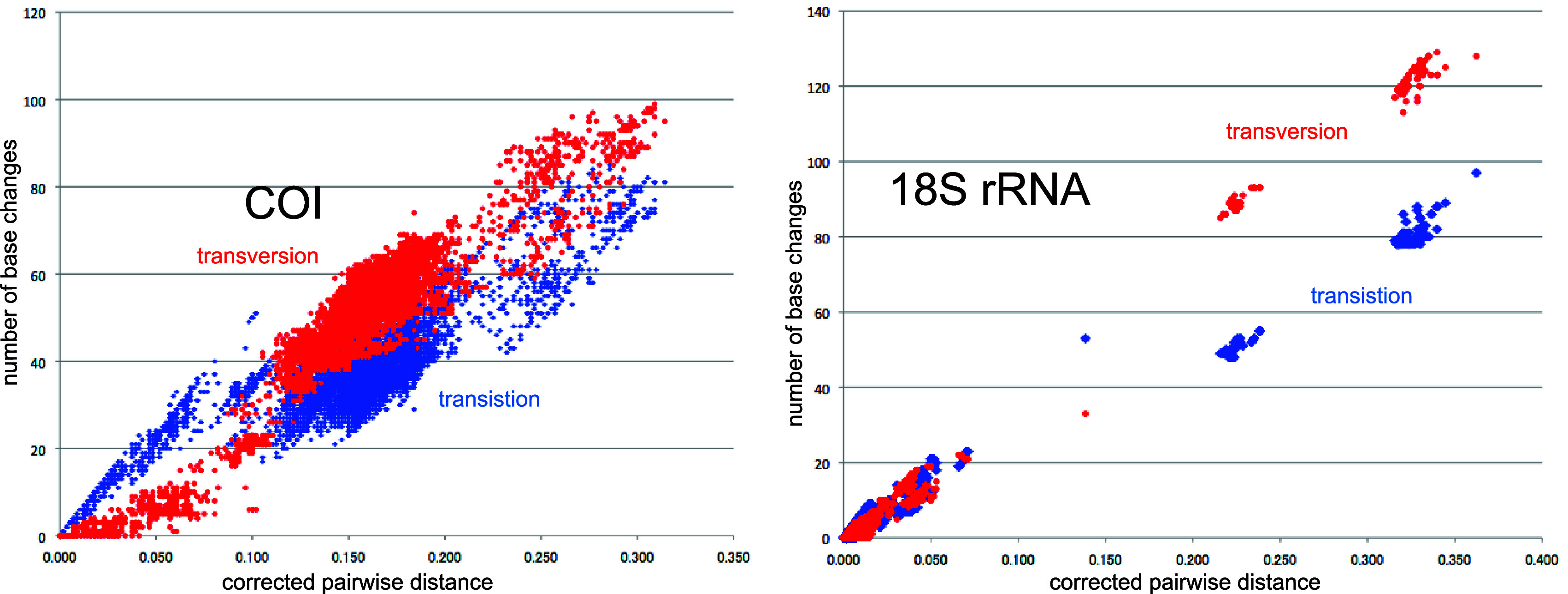
Number of base changes of transition and tansversion vs corrected pairwide distance diagram for whole mitochondrial gene.

The inset in Figures 1– 3 shows that the base substitution rate was relatively slow until the Quaternary higher rate. To evaluate whether the slow rate reflected saturation, we examined the relation between pairwise distance and number of transition or transversion for each gene, using the MEGA5 function ( Tamura *et al.* 2011; Figure 4).

### Fossil and geological event calibrations by BEAST v1. X

Calibrations points are shown on minimum age trees in Figures 1– 3, and these dates were input in “Priors” in BEAUti as noted above; they are summarized below ( Table 2). As noted above, corresponding ingroup species were included in ingroup taxa (= leaf node taxa in a specific clade) by Taxon Set on the Taxa screen in BEAUti.

Fossil calibrations are after Johnson *et al.* (2018) and Moulds (2018) ( Table 2; Figures 1– 3). For these fossil calibrations, some are based on radio-isotopic dating of the fossil-bearing strata, whereas others are based on biostratigraphy assigned to an age/stage on the geologic time scale, for which absolute age ranges are generally based on radio-isotopic dates of associated strata in key global localities. This time scale has been standardized by the International Commission on Stratigraphy (ICS) ( www.stratigraphy.org) and the most recent version of the time scale is available at http://www.stratigraphy.org/index.php/ics-chart-timescale, and the explanatory paper related to the generation of the time scale is Cohen *et al.* (2013).

Calibration points Q1 to Q6 and Q8 to Q12 are after our geological event calibration that adopts a 1.55 Ma date ( Osozawa *et al.* 2012). This geologic event calibration was used in previous studies of *Platypleura* cicadas ( Osozawa *et al.* 2017a) and four cicada groups ( Osozawa *et al.* 2021a).

The specific calibration points are as follows: tMRCA of *Mogannia minuta* (Q1), *M. hebes* (Q2), *Cryptotympana facialis* (Q3), dark winged *Platypleura* (Q4) and right winged *Platypleura* (Q5; Osozawa *et al.* 2017a), *Graptopsaltria nigrofuscata* + *G. bimaculata* (Q6), *Meimuna kuroiwae* (Q8) *, M. oshimaensis + M. iwasakii* + *M. goshizana* (Q9), *Tanna japonensis* + *T. japonensis ishigakiana* + *T. sozanensis* + *T.* sp. (Q10) *Euterpnosia chibensis* + *E. chibensis daitoensis* + *E. chibensis okinawana* (Q11), *E. iwasakii* + *E. viridifrons* + *E. olivacea* + *E. gina* + *E.* sp. (Q12): The date of the geological event, which records the isolation of the Ryukyu Islands from the Chinese mainland by the opening of the Okinawa trough that began (i.e., islands had separated from mainland and each other by this time) at 1.55 ± 0.15 Ma ( Osozawa *et al.* 2012). The age assignment is from multiple biostratigraphic and radio-isotopic ages from the oldest marine strata on the landward side of the islands as well as the sides facing other islands, so that the age of such strata constrains the physical separation of the islands from the mainland and each other. There is no geologic evidence for land bridges that could have aided dispersal in the Ryukyu Islands.

Calibration point Q7 ( *Meimuna opalifera*) is distinct from the above 1.55 ± 0.15 Ma event calibration. Hachijo oceanic island is a part of the Izu volcanic arc, and we recently estimated the emergence time of Hachijo-jima as an island at 0.24 Ma ( Osozawa *et al.* 2021b). This date is applicable for crown *Meimuna opalifera* on the Hachijo-jima + the Japan-Tokara islands (= Stem *Meimuna opalifera* on Hachijo-jima).

With the assumption that the oldest fossil age is equivalent tMRCA (= minimum age), the specific fossil calibration points and associated dates are as follows:

Calibration point A1: Crown Hemiptera: Fossils of Aphidoidea were reported from the French Bundsandstein ( Szwedo & Nel 2011; Bashkuev *et al.* 2012) of Anisian age (244.5 ± 2.5 Ma).

A2: The oldest fossil Belostomatidae was reported from the Zagaje Formation, Poland ( Popov 1996) of Hettangian age (200.3 ± 1.0 Ma).

A3: Fossil Ledridae ( Zhang 1997) and fossil Cercopidae ( Hong 1982) were recovered from the Jehol Biota of northern China. The Jehol Biota horizon has been dated by the Ar-Ar method on associated silicic tuff at 130.7 ± 1.4 Ma ( He *et al.* 2006).

A4: Fossil Gerridae were recovered from French amber ( Perrichot *et al.* 2005) of Albian age (107 ± 6 Ma).

A5: Fossil Delphacidae were found in the Green River Formation, USA ( Grande 1980). Ar-Ar dating on silicic tuff within the formation yields ages of 53.5 – 48.5 Ma (weighted average age of 51.25 ± 0.31 Ma; Smith *et al.* 2003).

A6: Fossil Flatidae were found in the Maíz Gordo Formation, northwest Argentina ( Petrulevičius 2011) of Paleocene age (61 ± 5 Ma).

Calibration point B: Stem Tettigarctinae: Oldest fossil of Tettigarctinae were found in strata Dorset, England ( Whalley 1985) of Hettangian age (203.1± 1.0 Ma).

Calibration point C: Stem Derotettiginae: The preferred food of *Derotettix mendosensis* is Amaranthaceae in Argentina ( Simon *et al.* 2019), and this worldwide C4 plant was phylogenetically studied by Piirainen *et al.* (2017). This plant fossil was reported by Zucol *et al.* (2018), and the fossil-bearing horizon was dated by the Ar-Ar method at 49.512 ± 0.019 Ma (Eocene; Woodburne *et al.* 2014). However, fossil *Burmacicada protera* were found from Burmese amber ( Poinar & Kritsky 2011). Detrital zircons from the amber bearing matrix yielded a maximum depositional age U-Pb age of 98.79 ± 0.62 Ma, that was interpreted to closely approximate the actual depositional age on the basis of geologic relationships and associated fossils ( Shi *et al.* 2012). We applied this older date of Burmese amber for stem Derotettiginae or crown Cicadidae.

Calibration point D: Stem *Platypedia putnami* (= crown Tibicininae): Fossil *Platypedia primigenia* were found in the Florissant Formation, Colorado, USA, and the associated strata was dated by the Ar-Ar method at 35.15 ± 1.65 Ma ( Mcintosh *et al.* 1992). However, we used an older crown date for crown Tibicininae based on fossil *Davispia bearcreekensis* that were found in the Fort Union Formation, Montana, USA ( Cooper 1941). The age of the enclosing strata has been considered Thanetian in age (57.6 ± 1.6 Ma) ( Flores & Bader 1999). Crown Cryptotympanini: Fossil *Hadoa grandiose* were also found in the Florissant Formation, Colorado, USA, but this calibration generated an unreasonable tree and was not adopted.

Calibration point E: Crown Cicadettinae: *Paracicadetta oligocenica* ( Boulard & Nel 1990) were recovered from deposits of Céreste, France, and this famous fossil locality was considered to be of Ruperian age (31 ± 2.9 Ma; Ducreux *et al.* 1985).

Calibration point F: Stem *Lyristes plebejus*: Fossil *Lyristes* sp. were reported from Seifhennersdorf, Germany ( Tietz *et al.* 1998), and associated strata was dated by the K-Ar method as 30.44 ± 1.52 Ma ( Walther & Kvacek 2007).

Calibration point G: Crown *Cryptotympana*: Fossil *Cryptotympana incasa* and *C. miocenica* (G1), and also *Hyalessa lapidescens* (G2) were found in Shanwang, Shandong, China ( Zhang 1989; Zhang *et al.* 1994), and these strata are considered to be time correlative to the European MN5 mammalian unit (16.45 ± 0.45 Ma; Roček *et al.* 2011).

Calibration point H: Crown *Meimuna* spp.: Fossil *Meimuna protopalifera* were found in the Itamuro Formation, Tochigi, Japan ( Fujiyama 1969; Yoshikawa 2005), and the zircon fission track age of correlative terrestrial strata of the Nashino Formation of the Sendai area is 6.4 ± 0.4 Ma ( Fujiwara *et al.* 2008).

## Results

### Hemiptera minimum age tree ( Figure 1)

Our timetree spans a range as old as ca. 250 Ma, and there is no evidence of saturation of mutations ( Figure 4), suggesting our minimum age tree is robust and reliable.

Because the topology is concordant between Figures 1 and 2 (COI) and Figure 3 (COI + 16S rRNA), the following description follows Figure 2 with 352 specimens. Our analyses was concordant to the subfamily classification of Marshall *et al.* (2018), Łukasik *et al.* (2019), and Simon *et al.* (2019). Figures 1 and 2 also include data in Price *et al.* (2019) and Hill *et al.* (2021).

Hemiptera, including Cicadoidea, has a single common ancestor of 242.96 Ma, as calibrated by the 244.5 ± 2.5 Ma age reviewed above as A1. The dated tree of the outgroup Hemiptera calibrated by A1 to A6 was concordant to Johnson *et al.* (2018) and Misof *et al.* (2014).

In the Cicadoidea ingroup, Tettigarctidae was an old lineage that differentiated from Cicadidae at 200.63 Ma, as calibrated by 200.3 ± 1 Ma (calibration point B), so Tettigarctidae is essentially a living fossil that has persisted since 200.63 Ma. We estimated a date of the common ancestor of two extant species of *Tettigarcta tomentosa* (Tasmania) and *T. crinita* (southeast Australia) at 13.96 Ma, and the youngest fossil of Tettigarctinae was reported from the Aquitanian (21.735 ± 1.295 Ma), southern New Zealand ( Kaulfuss & Moulds 2015). However, Tettigarctidae includes 19 extinct genera according to Kaulfuss and Moulds (2015) and with many more genera according to Moulds (2018).

Simon *et al.* (2019) proposed a new subfamily Derotettiginae consisting of a single species of *Derotettix mendosensis*, which is a sister of the remaining Cicadidae species and the oldest lineage species in Cicadidae dated at 99.2 Ma, as calibrated by point C at 98.79 ± 0.62 Ma. Łukasik *et al.* (2018) showed such a basal lineage of *D. mendosensis* in Cicadidae.

Our timetree showed that Tibicininae is a sister of Tettigomyiinae + Cicadettinae + Cicadinae and differentiated at 66.15 Ma, and Tibicininae started differentiation at 57.31 Ma, as calibrated by point D at 57.6 ± 1.6 Ma. Tettigomyiinae is a sister of Cicadettinae and differentiated at 35.46 Ma, Tettigomyiinae + Cicadettinae is a sister of Cicadinae differentiated at 40.57 Ma. Cicadettinae started differentiation at 30.85 Ma, as calibrated by point E at 31 ± 2.9 Ma. Cicadinae started differentiation at 38.25 Ma. Differentiation of Tettigomyiinae + Cicadettinae took place simultaneously after 35.46 Ma.

A single common ancestor of Cicadidae except Derotettiginae started differentiation and speciation into Tibicininae, Tettigomyiinae, Cicadettinae, and Cicadinae at 66.15 Ma. Although the pre-Miocene fossil Cicadidae collectively include ten extinct genera, comprising *Davispia* and *Lithocicada* for Tibicininae, *Paracicadetta*, *Paleopsalta*, *Minyscapheus*, and *Miocenoprasia* for Cicadettinae, and *Burmacicada, Camuracicada*, *Tymocicada*, *Dominicicada* for Cicadinae, the remaining 23 genera post-Oligocene fossil cicadas are extant ( Moulds 2018). Cicadidae, consisted of only one species but coexisted with a Tettigarctidae species between 200.63 and 66.15 Ma, and cicada biodiversity was extremely low during this period except for extinct species and *D. mendosensis.*

In the Cicadettinae major clade, each tribe constitutes a distinct clade. In the Cicadinae major clade, apart from older five tribe clades containing only one specimen, six tribe clades of Platypleurini, Cryptotympanini, Psithyristriini, Dundubiini + Cosmopsaltriini, Polyneurini + Sonatini, and Leptopsaltriini + Gaeanini are recognized. Discrepancies are addressed by reconsideration of taxonomy in the discussion.

The geologic calibration points Q1 to Q12 at 1.55 ± 0.15 Ma (and 0.24 Ma) apply to multi furcations that were recognized for *Mogannia minuta* and other cicadas endemic to in the Ryukyu Islands and Taiwan (and in Hachijo-jima) as noted above. Each island or island group population was mostly genetically distinct, endemic, and cryptic, as shown for *Platypleura* in Osozawa *et al.* (2017a). This also applies to *Meimuna opalifera* on Hachijo oceanic island ( Osozawa *et al.* 2021a, b). However, note that some cicadas were accidentally dispersed by super typhoons up to 1,000 km in modern and ancient times including *Meimuna boninensis* ( Osozawa *et al.* 2021a).

### Inconsistent cicada base substitution rate ( Figures 1– 3 insets)

Comparing base substitution rate vs age shows that the rate has not been constant; the rate appears to have exponentially increased into the Holocene. The data points, approximate curve, and associated equation are shown on the insets of Figures 1– 3. The curves and associated rates are similar for analyses based on COI alone ( Figures 1 and 2 insets), and combined COI + 18S rRNA ( Figure 3 inset).

Figure 4 shows that even mitochondrial COI gene with rapid base substitution rate ( Osozawa *et al.* 2017a) is never saturated toward the ancient time up to ca. 250 Ma.

## Discussion

### Taxonomic implications from the dated tree

Tibicininae is solely from North and South America with an exceptional occurrence from the Mediterranean region, but absent from Asia and Africa (+ Australia). The stem age is estimated at 66.15 Ma ( Figures 1 and 2), and if we assume that Tibicininae was generated by vicariance its differentiation may have been influenced by the formation of the Atlantic Ocean. Marine magnetic anomalies on the Atlantic Ocean floor can be used to ascertain spreading history and separation of continents that resulted from this spreading. The configuration at Chron34 (84Ma) after the Cretaceous magnetic quiet zone (long normal polarity epoch; superchron K-T at 118-84 Ma) was shown by Moulin *et al.* (2010), and the south Atlantic Ocean spread over 500 km (minimum distance between Africa and South America) at Chron 34 (84 Ma). The date of 84 Ma can be considered to be a starting date of continent level vicariance, which may have triggered the Tibicininae differentiations relative to especially Cicadinae shown in Figures 1 and 2.

*In Cicadettinae,* Prasiini is a sister of Cicadettini. *Muda kuroiwae* in Prasiini ( Hayashi & Saisho 2011) is endemic and restricted to Okinawa-jima and Kume-jima, and represents as a sister of the similar species of *Katoa taibaiensis* on the Chinese mainlamd.

In the Moganniini clade, *Nipponosemia terminalis* (Matsumura 1913) (synonym: *Vagitanus terminalis*) is a sister of *Mogannia* spp. *N. terminalis* has been documented from the Yaeyama islands and Miyako-jima (endangered and protected), Ryukyu, and Taiwan ( Figure 2 from the Taiwan specimen; another species of *N. virecens* is known from the Kaoshun peninsula, southern most Taiwan; Lee & Hayashi 2004), but Yang & Wei (2013) reported *N. terminalis* and other three *Nipponosemia* from China. A detailed phylogenetic study for these *Nipponosemia* species would be useful. The genitalia and morphological character are similar to *Mogannia* ( Hayashi & Saisho 2011), concordant to the sister relationship with *Mogannia.* See Osozawa *et al.* (2021a) for the *Mogannia minuta* vicariant speciation on the Ryukyu Islands and the accidental typhoon dispersals in recent and also ancient times. *Mogannia hebes* in northern Taiwan and in southern China has sister relationship reflecting vicariance by the Taiwan strait (Osozawa *et al.* 2011), and this species in southern Taiwan was differentiated relative to the northern Taiwan species reflecting vicariance triggered by the physical barriers of the Yilan basin and Lanyang valley ( Osozawa *et al.* 2017b; http://kawaosombgi.livedoor.blog/?p=26 and others).

We combined East Asian *Platypleura* data after Osozawa *et al.* (2017a) with mostly African Platypleurini data excluding *Platypleura* after Price *et al.* (2019), and the terminal node of the East Asian *Platypleura* in the Platypleurini clade suggests the possibility of a Gondwanan origin and dispersal to Far East of Japan and Ryukyu, and Taiwan ( Price *et al.* 2019). See Osozawa *et al.* (2017a) for the *Platypleura* vicariant speciation (see below for the cryptic speciation) on the Ryukyu Islands.

*Tacua speciosa* (Tacuini) represents the basal lineage of Cryptotympanini concordant with Marshall *et al.* (2018). In the Cryptotympanini clade, *Auritibicen* in Japan is the basal lineage, and *Lyristes plebejus* (synonym: *Tibicen plebejus*) in Croatia is the next. Asian Cryptotympana species is a sister of North American *Noetibicen* species ( Hill *et al.* 2015). Intercontinental dispersal by way of a Bering land bridge during Oligocene to Miocene climatic optima ( Wu *et al.* 2015) was proposed for Papilionoidea butterflies feeding on Magnoliidae.

*Zammara smaragdina*, Costa Rica, represents the basal lineage of the remaining major clades that are paraphyletic each other. *Distantalna splendida*, renamed from *Tosena splendida* by Boulard (2009; referred in Hill *et al.* 2015), is represented by Tosenini, as a next basal lineage, distinct from another Tosenini of *Tosena melanopteryx* in the Psithyristriini composite clade. *Tosena* (Tosenini) and *Pomponia* (Psithyristriini) has a sister relationship, and these are similar tribes (species level transfer may be needed; Duffels & Hayashi, 2006). *Pomponia backanensis*, northern Vietnam, was described by Pham *et al.* (2015). *Pomponia linearis* on the Yaeyama islands and Taiwan, mildly differentiated each other as cryptic species, was renamed *P. yayeyamana* based on Kato (1932). The original *P. linearis* was reported from primarily Indochina, and has been treated as the *P. linearis* complex, including cryptic Chinese and Indian populations ( Hayashi & Saisho 2011). *Unipomponia decem* (Psithyristriini?) was renamed *Pomponia decem* ( Lee & Sanborn 2010).

*Megapomponia* ( Lee & Sanborn 2010) was associated with the genera from Dundubiina ( Hill *et al.* 2021), and included in the Dundubiini clade. Oceanian Cosmopsaltriini is a sister of Asian Dundubiini, reflecting large scale vicariance driven by bio-geographic barrier of the Wallacea line, as well as endemism within the islands by Oceanian arc fragmentations ( Boer & Duffels 1996). In the Dundubiini clade, see Osozawa *et al.* (2021a) for the vicariant and cryptic speciation of *Meimuna kuroiwae* on the Ryukyu islands and the accidental typhoon dispersals in recent and ancient times (including *Meimuna boniensis* on the oceanic Bonin islands). See Osozawa *et al.* (2021a) for the vicariant and cryptic speciation of *Meimuna opalifera* on the oceanic Hachijo-jima island ( Osozawa *et al.* 2021b) by the accidental typhoon dispersal from the Japan continental islands. *Meimuna mongolica* in Korea and China is the basal lineage relative to the sympatric *M. opalifera.* *Meimuna oshimensis* endemic on the Amami and Okinawa islands (cryptic species), *Meimuna iwasakii* endemic on the Yaeyama islands and Taiwan (no specimen collected from Taiwan specimen; cryptic species), and *Meimuna goshizana* and *M. gakokizana*, other endemic species on Taiwan, were vicariantly speciated or adaptively radiated in Taiwan.

*Hyalessa* is in Sonatini was renamed from *Oncotympana* in the distinct Oncotympanini. *Hyalessa maculaticollis* in Japan and China ( Liu *et al.* 2018) is deeply differentiated. Sonatini is a sister of Polyneurini (once included in Tosenini; noted in Hayashi & Saisho 2011), and these constitute the Polyneurini + Sonatini clade. *Graptopsaltria nigrofuscata* in Japan and *G. bimaculata* on the Amami and Okinawa islands were vicariantly speciated.

*Terpnosia* cf. *graecina* was synonymised with *Leptopsaltriina* (discussed in Hill *et al.* 2021) constitutes the basal lineage with Thailand Leptopsaltriini in the Leptopsaltriini-Gaeanini major clade. *Kalabita operculata*, Malaysia, was a member of above-mentioned Platypleurini known to have diversified in Africa ( Price *et al.* 2019), but this Asian species is included in the Leptopsaltriini + Gaeanini clade. Trilar (2006) presented an adult photo of *K. operculata* showing that it lacks the pronotum that characterizes Platypleurini. Furthermore, the spectrogram - oscillogram of its song is similar to those of *Euterpnosia* spp., Leptopsaltriini, shown in Hayashi and Saisho (2011). Platypleurini both in African and Asia are monophyletic as noted above, and an exception is unreasonable. Hill *et al.* (2021) transferred *Kalabita* Moulton, 1923 from Platypleurini to Leptopsaltriini. *Tosena paviei* is a member of Tosenini, but is a sister of *Callogaeana guanxiensis* in Gaeanini, and renamed as *Vittagaeana paviei* in Gaeanini by Hill *et al.* (2021). Both the species are from Vietnam, and constitute a Gaeanini clade with another Vietnam species of *Balinta* cf. *tenebricosa* of Gaeanini. An interesting result is that Gaeanini including *T. paviei* is not monophyletic, and paraphyletic in the Leptopsaltriini major clade. Hill *et al.* (2021) showed wing phenotypes of Gaeanini- Tosenini, which are distinct from phenotypes of Leptopsaltriini. Note that *Gaeana* (Gaeanini) is also a sister of *Tanna* (Leptopsaltriini) with different wing and chest phenotypes. *Tanna japonensis* is differentiated between Japan and the isolated population on the Amami-Oshima island, and further differentiated from the isolated *Tanna japonensis ishigakiana* population on Ishigaki-jima island. Taiwan yields *Tanna sozanensis* (sister of *T. japonensis ishigakiana*) and the other seven *Tanna* species ( Chen 2011), might have adaptively radiated within the island. *Tanna* in China is a sister of *T. sozanensis.* According to Hill *et al.* (2021), *Cicadmallus micheli* is characterised by an unusual ‘hammer-head’ morphology but otherwise bears morphological relationships to Leptopsaltriini, and represents the basal lineage of Indochina *Terpnosia* ( Thai & Yang 2009) and East Asian *Euterpnosia* of the Leptopsaltriini clade. See Osozawa *et al.* (2021a) for the vicariant and cryptic speciation for *Euterpnosia* for the northern population on Japan-Amami-Okinawa and the southern population on Yaeyama-Taiwan, as well as accidental typhoon dispersal in ancient times ( *Euterpnosia chibensis daitoensis* on the oceanic Daito islands from Tokuno-shima continental island). Taiwan yields *Euterpnosia gin*a, *E. olivacea*, *E. viridifrons*, and other 12 *Euterpnosia* species ( Chen 2011), and may have adaptively radiated within the island.

### Recently increased cicada biodiversity

Hemipteroid insects of Psocodea, Thysanoptera, and the subject of this study, Hemiptera, include 120,000 described species which comprise over 10% of known insect diversity; they date back to 400 Ma (Hemiptera: 300 Ma; Johnson *et al.* 2018). Johnson *et al.* (2018) estimated that differentiation into species took place primarily in the Cretaceous, including Cercopoidea, Gerriidea, Flatidae, and Cicadoidea, which are common to our analyses. However, they analyzed only two to nine taxa, in contrast to the 344 taxa of Cicadoidea and 8 taxa for other Hemiptera of our analyses. Misof *et al.* (2014) estimated mostly pre-Paleogene dates of differentiation into species including Cercopoidea, Aphididae, and Delphacidae, concordant with our analyses, with less than 13 taxa analyzed. Their higher-level phylogeny suggested long branches and an old lineage of each super family species concordant with ours, but did not suggest the geologically recent increase in insect diversity apparent from our analyses of 352 Hemiptera taxa ( Figure 2).

In Figure 2, ingroup Cicadidae, excluding Derotettiginae, underwent extensive differentiation into 341 taxa after 66.15 Ma, mostly after 40.57 Ma, leading to increasing biodiversity of Cicadidae, although Price *et al.* (2019) suggested that number of lineages saturated in the Pleistocene. Cicadidae consisted of only two species including *D. mendosensis* between 99.2 and 66.15 Ma, although Cicadidae contains many extinct species that remain to be identified as fossils ( Moulds 2018).

Cryptic species on each island of Ryukyu chain are typical examples of increased biodiversity. For example, *Platypleura kaempferi* in the Amami and Okinawa islands has light colored wings, contrasting with dark colored wings in Japan-Korea-China and Taiwan, and the clades are distinct from each other ( Osozawa *et al.* 2017a). *P. kaempferi* is not a single species but includes at least two cryptic species of light or dark winged *Platypleura.* Cicadas calibrated by other Quaternary calibration points include cryptic species, which also contributed to increasing biodiversity. The Okinawa trough is currently spreading (widening) and the Ryukyu islands are separating from the Chinese mainland. Accordingly vicariant speciation and radiation is in progress, which is also contributing increasing biodiversity. On the Chinese mainland, *Hyalessa maculaticollis* and *Platypleura hilpa* extensively radiated to form cryptic species ( Liu *et al.* 2018, 2020).

### Exponentially increased base substitution rate as a factor of Hemiptera diversity, and their possible causes

Figures 1– 3 insets show a large range of base substitution rates for different time periods, at variance with the constant molecular clock hypothesis (relatively constant rate over time; Ho 2008). The trend in base substitution rates shows an exponential increase into the Holocene.

Such an increase in base substitution rate was first shown for taxa such as primates by Ho *et al.* (2005) who showed that a Quaternary calibration date resulted in a more a rapid base substitution rate than that associated with an older calibration date. They employed an older version of BEAST (v1. 3; Drummond & Rambaut 2003) that required repeated runs, applying a date at each calibration point. In contrast, BEAST v1. X, used in our analyses, can simultaneously apply multiple calibration points, as we have done using dates ranging from the Triassic to the Quaternary. As a result, the calculated increasing rate of base substitution in our analyses is not an artifact of a Quaternary calibration, but is constrained by multiple age calibrations across a wide range of geologic time. Therefore, although the base substitution rate trendlines and associated equations of Ho *et al.* (2005) are similar to ours, their timetrees do not reflect the changing of base substitution rates through time, but rather reflect a constant base substitution rate as if constrained by a strict molecular clock. A similar analysis was done for beetles in the Aegean region by Papadopoulo *et al.* (2010).

The increasing base substitution rate is apparently associated with the recently increasing cicada diversity, expansion, and radiation (in Figure 2 timetree) that started at 40.57 Ma. The rapid diversification of Delphacini is linked to shifts in host plants, particularly from C3 to C4 grasses within the Poaceae family ( Urban *et al.* 2010). The timing of the most rapid diversification coincides with Quaternary environmental change, marked by the start of glacial-inter glacial cycles. The initiation of Quaternary glaciations may have been triggered by rapid expansion of land grasses (Poales), that led to increased carbon fixation that decreased atmospheric CO _2_ concentrations, because of the high efficiency of CO _2_ fixation of such C4 plants ( Sage 2004; Taira 2007). C4 Poales appeared and began diversification during the Oligocene (23 – 33.9 Ma) based on molecular clock approach, and after 14.5 Ma based on fossil evidence ( Sage 2004). We estimated 20.35 Ma by our Angiospermae timetree, that also employed BEAST v1. X with robust plant fossil calibrations ( Osozawa *et al.* 2021c).

Food plants of *D. mendosensis* are, however, C4 dicots of Amaranthaceae (see figure 9 in Sage 2004) and Chenopodiaceae in degraded salt-plain habitats in arid regions of central Argentina ( Simon *et al.* 2019). These dicot fossils and C4 monocot fossils of Poales grass (Chloridoidae) were reported from the Eocene in Patagonia by Zucol *et al.* (2018), and the fossil horizon was dated by the Ar-Ar method at 49.512 ± 0.019 Ma ( Woodburne *et al.* 2014). The C4 photosynthetic pathway began at ca. 50 Ma in South America, earlier than elsewhere. For Chloridoideae, however, transition from C3 to C4 photosynthesis occurred in the Oligocene (23~33.9 Ma) as reported by Christin *et al.* (2008), consistent with our estimate for C4 dicots at 31.92 Ma ( Osozawa *et al.* 2021c), whereas Sage (2004) suggested a fossil date at 14.5 Ma as noted above.

The trigger of increasing biodiversity may have been the generation and radiation of C4 plants and development of grass lands on the Earth since the Oligocene or perhaps more definitively since middle Miocene, by decreasing atmospheric CO2 concentrations. This may have led to the start of Quaternary ice ages and resultant adaptive radiation and increasing base substitution (≒ mutation) rates. Thus, biologic activity, including spreading C4 grasses may have significantly impacted Earth's environment.

## Data availability

Sequence data in Table 1 are found in GenBank/DDBJ by incorporating the accession number. The xml file generated by BEAUti for running the BEAST platform software contains all the utilized sequence data and can be obtained upon email request to the senior author.
