# Supplementary material for: Cicada minimum age tree: Cryptic speciation and exponentially increasing base substitution rates in recent geologic time
Source: F1000Res. 2025 Feb 14;11:308. Originally published 2022 Mar 14. [Version 5] doi: 10.12688/f1000research.76068.5 (PMC11564946; doi:10.12688/f1000research.76068.5)
Supplement: Peer review history

# Reviewer report: Reviewer response for version 5

**DOI:** 10.5256/f1000research.177914.r366525

**Published:** 2025-03-03

**Authors:** Christopher H Dietrich

**Affiliations:** 1 University of Illinois Champaign, Champaign, Illinois, USA

The new version of the manuscript addresses my prior concerns so I now think it is acceptable in its present form.

 -CD

Is the work clearly and accurately presented and does it cite the current literature?

Partly

If applicable, is the statistical analysis and its interpretation appropriate?

Yes

Are all the source data underlying the results available to ensure full reproducibility?

Yes

Is the study design appropriate and is the work technically sound?

Yes

Are the conclusions drawn adequately supported by the results?

Yes

Are sufficient details of methods and analysis provided to allow replication by others?

Yes

Reviewer Expertise:

NA

I confirm that I have read this submission and believe that I have an appropriate level of expertise to confirm that it is of an acceptable scientific standard.

# Reviewer report: Reviewer response for version 4

**DOI:** 10.5256/f1000research.170027.r354651

**Published:** 2025-01-29

**Authors:** Christopher H Dietrich

**Affiliations:** 1 University of Illinois Champaign, Champaign, Illinois, USA

This interesting paper provides a detailed timetree for cicadas, combining several new minimum age calibrations for recent speciation events in Asia to some calibrations for some older divergences published previously. The methods are described in detail and the conclusions seem justified. Of particular interest is the exponential increase in nucleotide substitution rates for the gene regions used to estimate the timetree. The main remaining problem with the manuscript is that it needs considerable editing to improve the English and clarify some points that are not currently clear as written, e.g., in the abstract it is stated "The Cicadinae clade consists of specific tribes with paraphyletic relationship"; does this mean that the tribes are each paraphyletic, some tribes gave rise to others, or something else? Earlier in the abstract the statement "Tettigarctidae had a cicada basal lineage as old as 200.63 Ma"; the meaning is again unclear but presumably this could be stated more precisely as "Tettigarctidae are sister to the remaining cicadas..."

Is the work clearly and accurately presented and does it cite the current literature?

Partly

If applicable, is the statistical analysis and its interpretation appropriate?

Yes

Are all the source data underlying the results available to ensure full reproducibility?

Yes

Is the study design appropriate and is the work technically sound?

Yes

Are the conclusions drawn adequately supported by the results?

Yes

Are sufficient details of methods and analysis provided to allow replication by others?

Yes

Reviewer Expertise:

Systematics, taxonomy, paleontology and phylogenomics of Auchenorrhyncha.

I confirm that I have read this submission and believe that I have an appropriate level of expertise to confirm that it is of an acceptable scientific standard, however I have significant reservations, as outlined above.

# Reviewer report: Reviewer response for version 4

**DOI:** 10.5256/f1000research.170027.r330173

**Published:** 2024-11-14

**Authors:** Jacek Szwedo

**Affiliations:** 1 University of Gdańsk, Gdańsk, Poland

The aims of research and methods are adequate. The results results are presented accurately and with justified conclusions. These are well supported by the provided data, selected calibration points and results, and provided tables and figures.

Is the work clearly and accurately presented and does it cite the current literature?

Partly

If applicable, is the statistical analysis and its interpretation appropriate?

Partly

Are all the source data underlying the results available to ensure full reproducibility?

Yes

Is the study design appropriate and is the work technically sound?

Yes

Are the conclusions drawn adequately supported by the results?

Partly

Are sufficient details of methods and analysis provided to allow replication by others?

Yes

Reviewer Expertise:

fossils, phylogeny, palaeoecology

I confirm that I have read this submission and believe that I have an appropriate level of expertise to confirm that it is of an acceptable scientific standard.

# Reviewer report: Reviewer response for version 3

**DOI:** 10.5256/f1000research.165295.r292455

**Published:** 2024-07-25

**Authors:** Jacek Szwedo

**Affiliations:** 1 University of Gdańsk, Gdańsk, Poland

There are several new data and interpretations concerning the classification and fossil record of cicadas and Hemiptera s whole - see attached file.

 Main issue is the selection and estimation of age taken for taxa used for calibration, these are to be revised and updated according to current data, classification proposals or if other than the oldest available taxa selected the reasons should be clearly explained. In my opinion the stem and crown groups should be clearly stated and pointed which are analysed and then discussed. The text is not easy to read, especially for those not familiar with numerous existing phylogenetic, classifications and relationships hypotheses. The better calibration points selection, and use of new data (e.g. Bucher et al. 2023, Jiang et al. 2024) can change the received topologies. There are several taxonomic decisions which are not clearly explained or references, e.g. transfer of *Burmacicada protera* from Cicadinae to Derotettiginae. Interpretation and discussion of data and results must be more detailed - the Clypeata is the only extant clade of extremely diversified Cicadomorpha, with most of the lineages gone extinct, this leads to misunderstandings of relationships based exclusively on molecular data, as these are available only for crown, extant, groups. This reservation must be taken for higher taxa especially. A comparison with evolutionary traits observed in Delphacidae planthoppers (Urban et al 2010 [Ref-11]) closely related to Poaceae can enrich the discussion and maybe similar phaenomena can be found and discussed. 

 Please refer to the following attachment for my additional comments on your article submission: https://f1000research.s3.amazonaws.com/linked/665193.Jaeck-Reviewer_attachment.pdf

Is the work clearly and accurately presented and does it cite the current literature?

Partly

If applicable, is the statistical analysis and its interpretation appropriate?

Partly

Are all the source data underlying the results available to ensure full reproducibility?

Yes

Is the study design appropriate and is the work technically sound?

Yes

Are the conclusions drawn adequately supported by the results?

Partly

Are sufficient details of methods and analysis provided to allow replication by others?

Yes

Reviewer Expertise:

fossils, phylogeny, palaeoecology

I confirm that I have read this submission and believe that I have an appropriate level of expertise to confirm that it is of an acceptable scientific standard, however I have significant reservations, as outlined above.

## References

[1] : Mesozoic evolution of cicadas and their origins of vocalization and root feeding. *Nat Commun* .2024;15(1) : 10.1038/s41467-023-44446-x 376 10.1038/s41467-023-44446-x 38191461 PMC10774268

[2] : Phylogeny and diversification of planthoppers (Hemiptera: Fulgoromorpha) based on a comprehensive molecular dataset and large taxon sampling. *Mol Phylogenet Evol* .2023;186: 10.1016/j.ympev.2023.107862 107862 10.1016/j.ympev.2023.107862 37331454

[3] : Time-traveling through fossil planthopper tegmina in the Paleozoic and Mesozoic eras (Insecta: Hemiptera: Fulgoromorpha). *Palaeoentomology* .2024;7(1) : 10.11646/palaeoentomology.7.1.1 1-67 10.11646/palaeoentomology.7.1.1

[4] : Toward a New Classification of Planthoppers Hemiptera Fulgoromorpha: 1. What Do Fulgoridiidae Really Cover?. *Annales Zoologici* .2022;72(4) : 10.3161/00034541ANZ2022.72.4.011 10.3161/00034541ANZ2022.72.4.011

[5] : Toward a new classification of planthoppers Hemiptera Fulgoromorpha: 2. Higher taxa, their names and their composition. *Zootaxa* .2023;5297(4) : 10.11646/zootaxa.5297.4.5 562-568 10.11646/zootaxa.5297.4.5 37518779

[6] : Revision of the Hylicellidae of the Late Triassic of Queensland (Hemiptera: Cicadomorpha: Hylicelloidea). *Zootaxa* .2020;4790(3) : 10.11646/zootaxa.4790.3.7 zootaxa.4790.3.7 10.11646/zootaxa.4790.3.7 33056698

[7] : The Middle Triassic Lagerstätte of Monte San Giorgio reveals the oldest lace bugs (Hemiptera: Tingidae): Archetingis ladinica gen n. sp n. *Rivista Italiana di Paleontologia e Stratigrafia* .2018; Reference source

[8] : The earliest true bugs and aphids from the Middle Triassic of France (Hemiptera). *rej* .2011;19(1) : 10.15298/rusentj.19.3.04 179-182 10.15298/rusentj.19.3.04

[9] : When did the ancestor of true bugs become stinky? Disentangling the phylogenomics of Hemiptera-Heteroptera. *Cladistics* .2019;35(1) : 10.1111/cla.12232 42-66 10.1111/cla.12232 34636080

[10] : The unity, diversity and conformity of bugs (Hemiptera) through time. *Earth and Environmental Science Transactions of the Royal Society of Edinburgh* .2016;107(2-3) : 10.1017/S175569101700038X 109-128 10.1017/S175569101700038X

[11] : Evolution of Delphacidae (Hemiptera: Fulgoroidea): combined-evidence phylogenetics reveals importance of grass host shifts. *Systematic Entomology* .2010;35(4) : 10.1111/j.1365-3113.2010.00539.x 678-691 10.1111/j.1365-3113.2010.00539.x

# Response

**Published:** 2024-08-05

**Authors:** Soichi Osozawa

**Affiliations:** Tohoku University, Japan

Reviewer Report

 2 Views

 copyright

 25 Jul 2024 | for Version 3

 Jacek Szwedo, State Key Laboratory of Palaeobiology and Stratigraphy, Nanjing Institute of Geology and Palaeontology, Chinese Academy of Sciences, Nanjing, China;  University of Gdańsk, Gdańsk, Poland

 format_quote Cite this report

 speaker_notes Responses(0)

 Approved With Reservations

 There are several new data and interpretations concerning the classification and fossil record of cicadas and Hemiptera s whole - see attached file.

 ---NOT readable.

 Main issue is the selection and estimation of age taken for taxa used for calibration, these are to be revised and updated according to current data, classification proposals or if other than the oldest available taxa selected the reasons should be clearly explained. In my opinion the stem and crown groups should be clearly stated and pointed which are analysed and then discussed. The text is not easy to read, especially for those not familiar with numerous existing phylogenetic, classifications and relationships hypotheses. The better calibration points selection, and use of new data (e.g. Bucher et al. 2023, Jiang et al. 2024) can change the received topologies. There are several taxonomic decisions which are not clearly explained or references, e.g. transfer of *Burmacicada protera* from Cicadinae to Derotettiginae. Interpretation and discussion of data and results must be more detailed - the Clypeata is the only extant clade of extremely diversified Cicadomorpha, with most of the lineages gone extinct, this leads to misunderstandings of relationships based exclusively on molecular data, as these are available only for crown, extant, groups. This reservation must be taken for higher taxa especially. A comparison with evolutionary traits observed in Delphacidae planthoppers (Urban et al 2010 [Ref-11]) closely related to Poaceae can enrich the discussion and maybe similar phaenomena can be found and discussed.

 Thank you for review.

 Bucher et al. (2023) provided the age of Cicadoidea fossils in Burmage amber as 98.79 Ma. I have used this age as calibration point C, representing the stem age of Derotettiginae (i.e., the stem of Cicadoidea).

 Bucher et al. (2023) and other studies provide estimated ages based on node dating, which are not suitable for the present fossil age calibration. Stem and crown ages should be determined using fossil evidence. Jiang et al. (2024) and other recommended articles may include such indirect ages, but I used geologically robust fossil ages, including those from Burmese amber, for calibration.

 Transfer of Burmacicada protera from Cicadinae to Derotettiginae.

 --- The transfer of Burmacicada protera from Cicadinae to Derotettiginae was originally noted as Derotettiginae in Table 2.

 the Clypeata is the only extant clade of extremely diversified Cicadomorpha, with most of the lineages gone extinct, this leads to misunderstandings of relationships based exclusively on molecular data, as these are available only for crown, extant, groups.

 --- I have already included Cercopidae (Cicadomorpha, which comprises extant species other than those in Cicadoidea).

 “A3: Fossil Ledridae ( Zhang 1997) and fossil Cercopidae ( Hong 1982) were recovered from the Jehol Biota of northern China. The Jehol Biota horizon has been dated by the Ar-Ar method on associated silicic tuff at 130.7 ± 1.4 Ma ( He et al. 2006).”

 I cited Urban et al (2010) at the end of discussion.

 “The rapid diversification of Delphacini is linked to shifts in host plants, particularly from C3 to C4 grasses within the Poaceae family (Urban et al., 2010).”

 Please refer to the following attachment for my additional comments on your article submission: https://f1000research.s3.amazonaws.com/linked/665193.Jaeck-Reviewer_attachment.pdf 

 Note that the data in the attachment may be lost or unreadable.

 Is the work clearly and accurately presented and does it cite the current literature?

 Partly

 Is the study design appropriate and is the work technically sound?

 Yes

 Are sufficient details of methods and analysis provided to allow replication by others?

 Yes

 If applicable, is the statistical analysis and its interpretation appropriate?

 Partly

 Are all the source data underlying the results available to ensure full reproducibility?

 Yes

 Are the conclusions drawn adequately supported by the results?

 Partly

 References

 1. Jiang H, Szwedo J, Labandeira CC, Chen J, et al.: Mesozoic evolution of cicadas and their origins of vocalization and root feeding.Nat Commun. 2024; 15 (1): 376 PubMed Abstract | Publisher Full Text

 2. Bucher M, Condamine FL, Luo Y, Wang M, et al.: Phylogeny and diversification of planthoppers (Hemiptera: Fulgoromorpha) based on a comprehensive molecular dataset and large taxon sampling.Mol Phylogenet Evol. 2023; 186: 107862 PubMed Abstract | Publisher Full Text

 3. BUCHER M, GIGNOUX G, SZWEDO J, BOURGOIN T: Time-traveling through fossil planthopper tegmina in the Paleozoic and Mesozoic eras (Insecta: Hemiptera: Fulgoromorpha). Palaeoentomology. 2024; 7 (1): 1-67 Publisher Full Text

 4. Bourgoin T, Szwedo J: Toward a New Classification of Planthoppers Hemiptera Fulgoromorpha: 1. What Do Fulgoridiidae Really Cover?. Annales Zoologici. 2022; 72 (4). Publisher Full Text

 5. Bourgoin T, Szwedo J: Toward a new classification of planthoppers Hemiptera Fulgoromorpha: 2. Higher taxa, their names and their composition.Zootaxa. 2023; 5297 (4): 562-568 PubMed Abstract | Publisher Full Text

 6. Lambkin KJ: Revision of the Hylicellidae of the Late Triassic of Queensland (Hemiptera: Cicadomorpha: Hylicelloidea).Zootaxa. 2020; 4790 (3): zootaxa.4790.3.7 PubMed Abstract | Publisher Full Text

 7. Montagna M., Strada L., Dioli P. & Tintori A: The Middle Triassic Lagerstätte of Monte San Giorgio reveals the oldest lace bugs (Hemiptera: Tingidae): Archetingis ladinica gen n. sp n.Rivista Italiana di Paleontologia e Stratigrafia. 2018. Reference Source

 8. Shcherbakov D: The earliest true bugs and aphids from the Middle Triassic of France (Hemiptera). rej. 2011; 19 (1): 179-182 Publisher Full Text

 9. Wang YH, Wu HY, Rédei D, Xie Q, et al.: When did the ancestor of true bugs become stinky? Disentangling the phylogenomics of Hemiptera-Heteroptera.Cladistics. 2019; 35 (1): 42-66 PubMed Abstract | Publisher Full Text

 10. Szwedo J: The unity, diversity and conformity of bugs (Hemiptera) through time. Earth and Environmental Science Transactions of the Royal Society of Edinburgh. 2016; 107 (2-3): 109-128 Publisher Full Text

 11. URBAN J, BARTLETT C, CRYAN J: Evolution of Delphacidae (Hemiptera: Fulgoroidea): combined-evidence phylogenetics reveals importance of grass host shifts. Systematic Entomology. 2010; 35 (4): 678-691 Publisher Full Text

 Competing Interests

 No competing interests were disclosed.

 Reviewer Expertise

 fossils, phylogeny, palaeoecology

 I confirm that I have read this submission and believe that I have an appropriate level of expertise to confirm that it is of an acceptable scientific standard, however I have significant reservations, as outlined above.

# Reviewer report: Reviewer response for version 2

**DOI:** 10.5256/f1000research.154265.r260431

**Published:** 2024-04-18

**Authors:** Fan Song

**Affiliations:** 1 China Agricultural University, Beijing, China

This study primarily focused on estimating the divergence times of Cicadoidea. Compared to previous studies, the strength of this research lies in its inclusion of local data and data from enigmatic cicadas of the Ryukyu Islands. Exploring cicada biodiversity in the Ryukyu Islands and surrounding areas, as well as their historical evolution, is quite intriguing. A highlight of the paper is the addition of 92 new data points. Additionally, the discussion includes extensive content on cicada biodiversity and evolutionary history, which is very impressive. However, this manuscript still has some issues, such as poor readability of the figures.

 I think some of the reasonable suggestions from the previous reviewers haven't been addressed by the authors. Such as "A typo in Abstract: parapheletic -> paraphyletic.", "It would be better to replace "country" with "sampling locality" in the title of Table 1". I haven't seen evidence of them being addressed in the revisions.

 In addition to the above, I suggest the public sharing and presentation of intermediate data, such as alignment matrices, input files and output tree for BEAST. I appreciate and agree with the suggestion to provide a detailed description of the software analysis procedures. The materials and methods section in this manuscript reads more like a step-by-step tutorial rather than a description typically found in research articles. If a thorough description is desired, perhaps it would be more suitable to include it in the supplementary materials.

 The following comments elaborate some places where the improvements can be made.

1. There's confusion in the description. The DNA sequences used are COI and 18S rRNA. However, in both the ‘Materials and Methods’ and ‘Results sections’, the description of Figure 3 mentions COI and 16S rRNA as well as COI and 18S rRNA simultaneously.
2. The captions for Figures 1 and 3 lack explanations for the different coloured asterisks, especially the red pentagon and the meaning of the small branches on the right side of the images.
3. In Figures 1­–3, the scatter plot of base substitution rate needs to specify the data used for the X-axis and Y-axis. In the Materials and Methods, I believe it's important to explain the conceptual rationale behind the design of base substitution rate.
4. The estimated divergence time ranges mentioned in the manuscript should ideally maintain the same number of decimal places for consistency.

Is the work clearly and accurately presented and does it cite the current literature?

Partly

If applicable, is the statistical analysis and its interpretation appropriate?

Yes

Are all the source data underlying the results available to ensure full reproducibility?

Partly

Is the study design appropriate and is the work technically sound?

Yes

Are the conclusions drawn adequately supported by the results?

Yes

Are sufficient details of methods and analysis provided to allow replication by others?

Partly

Reviewer Expertise:

Insect Phylogeny; Genomics

I confirm that I have read this submission and believe that I have an appropriate level of expertise to confirm that it is of an acceptable scientific standard, however I have significant reservations, as outlined above.

# Reviewer report: Reviewer response for version 1

**DOI:** 10.5256/f1000research.80022.r184588

**Published:** 2023-08-02

**Authors:** David A Duchêne

**Affiliations:** 1 Center for Evolutionary Hologenomics, University of Copenhagen, Copenhagen, Capital Region of Denmark, Denmark

In this study, Osozawa and Wakabayashi examine the timing of the splits within the Cicadas using a Bayesian framework for phylogenetic analysis. The motivation for the study and data are well presented. However, the methods have large amounts of superfluous information that makes them appear confusing. Overall, the manuscript is going in a good direction, but requires substantial streamlining.

 My main comment is on the description of BEAST. For the purposes of this study, BEAST v1 and 2 should make very little difference. In fact, it would be surprising to find any difference in results whatsoever, unless the analyses failed to converge to stationarity. The differences highlighted by the authors, such as in the BEAUti interface, are not meaningful to the analyses. Therefore, the authors should consider removing any comparison between the software, and only mention BEAST once and very briefly in the methods.

 Far more important is the models chosen for analysis using BEAST. At the moment these details seem buried in between the discussion about the software versions. I suggest the authors replace any sections about the BEAST versions, and exclude BEAST from section headings, leaving simply a section on "Molecular dating". Importantly, the authors do not mention how they assessed convergence of the MCMC chain to stationarity (e.g., effective sample sizes, multiple runs, and visual assessment, or similar).

 There is substantial redundancy in the figures, which is confusing to the untrained eye. The authors should consider using a single main tree figure with the primary results and icons to indicate which analyses were congruent. This would make the study far more succinct and intuitive.

 The excessive emphasis on the software versions obscures the true emphasis of the study: Cicada evolution. The authors should consider re-framing the Abstract and Introduction to focus on the previous biological findings on the topic. The methodological considerations are secondary and should be left to the methods. Otherwise, the study appears to primarily focus on a debate about methods, which is not the case. The emphasis should be on the novel molecular and fossil data, which are the true major source of novel insights in the study.

Is the work clearly and accurately presented and does it cite the current literature?

Partly

If applicable, is the statistical analysis and its interpretation appropriate?

Yes

Are all the source data underlying the results available to ensure full reproducibility?

Yes

Is the study design appropriate and is the work technically sound?

Partly

Are the conclusions drawn adequately supported by the results?

Yes

Are sufficient details of methods and analysis provided to allow replication by others?

Yes

Reviewer Expertise:

Phylogenetic modelling, molecular evolution, molecular dating.

I confirm that I have read this submission and believe that I have an appropriate level of expertise to confirm that it is of an acceptable scientific standard, however I have significant reservations, as outlined above.

# Response

**Published:** 2023-08-06

**Authors:** Soichi Osozawa

**Affiliations:** Tohoku University, Japan

copyright

 02 Aug 2023 | for Version 1

 David A Duchêne

 Center for Evolutionary Hologenomics, University of Copenhagen, Copenhagen, Capital Region of Denmark, Denmark 

 In this study, Osozawa and Wakabayashi examine the timing of the splits within the Cicadas using a Bayesian framework for phylogenetic analysis. The motivation for the study and data are well presented. However, the methods have large amounts of superfluous information that makes them appear confusing. Overall, the manuscript is going in a good direction, but requires substantial streamlining.

*Thank you for your review and approval. I plan to compare MCMCTree to BEAST v1 and eagerly await the MCMCTree GUI for Mac. If you are involved in the development of MCMCTree, I appreciate your contributions to the field.*

 My main comment is on the description of BEAST. For the purposes of this study, BEAST v1 and 2 should make very little difference. In fact, it would be surprising to find any difference in results whatsoever, unless the analyses failed to converge to stationarity. The differences highlighted by the authors, such as in the BEAUti interface, are not meaningful to the analyses. Therefore, the authors should consider removing any comparison between the software, and only mention BEAST once and very briefly in the methods.

*Regarding the comparison to BEAST v2, I understand that it has undergone significant modifications and is distinct from v1, even though they share some developers. If you prefer, you may choose to use BEAST v1 for this study.*

 Far more important is the models chosen for analysis using BEAST. At the moment these details seem buried in between the discussion about the software versions. I suggest the authors replace any sections about the BEAST versions, and exclude BEAST from section headings, leaving simply a section on "Molecular dating". Importantly, the authors do not mention how they assessed convergence of the MCMC chain to stationarity (e.g., effective sample sizes, multiple runs, and visual assessment, or similar).

*Title "Molecular dating" may be a suggestion to avoid negative review for us, but I would like to describe details.*

*It is important to emphasize that a larger sample size and genome size, along with an increased MCMC chain, is unrelated to dating and calibrating the tree.*

*The MCMC chain is retained at its default settings.*

 There is substantial redundancy in the figures, which is confusing to the untrained eye. The authors should consider using a single main tree figure with the primary results and icons to indicate which analyses were congruent. This would make the study far more succinct and intuitive.

*I have simplified Fig. 1 to the best of my ability.*

*The node data, including base substitution rates, may be visualized in FigTree, created also by MCMCTree.*

 The excessive emphasis on the software versions obscures the true emphasis of the study: Cicada evolution. The authors should consider re-framing the Abstract and Introduction to focus on the previous biological findings on the topic. The methodological considerations are secondary and should be left to the methods. Otherwise, the study appears to primarily focus on a debate about methods, which is not the case. The emphasis should be on the novel molecular and fossil data, which are the true major source of novel insights in the study.

*I am emphasizing the increase in base substitution rate towards the present. To achieve this, the fossil dating process in BEAST v1 is thoroughly described. However, it is important to note that dating by MCMCTree and BEAST v2 does not align with our results, possibly due to the impact of maximum age assumptions.*

# Reviewer report: Reviewer response for version 1

**DOI:** 10.5256/f1000research.80022.r179259

**Published:** 2023-06-30

**Authors:** Sungsik Kong

**Affiliations:** 1 University of Wisconsin-Madison, Madison, Wisconsin, USA

The main objective of this study is to present a time-calibrated tree of cicada species reconstructed using BEAST v1.X. The trees contain crypic cicada species from Ryukyu islands in addition to the existing data presented in the previous studies. The authors show improved taxonomy of some of the major cicada groups. Moreover the authors suggest an exponential increase of base substitution rate in recent geologic time. While the content of the manuscript is interesting, it requires substantial improvement and is not ready for indexing. The following comments elaborate some places where the improvements can be made.

- In general, the manuscript is not easy to understand. It is understandable that English may not be the authors' first language, however, an academic manuscript must be written clearly. For example, the last sentence of Abstract seems to be not connecting with the previous sentences. The first sentence of Introduction is excessively long. 
- In figure 1, is is not clear what it means by "maximum" COI sequence. It is not clear what is the inserted figure. I assume it is the plot on the left side with white background. These are also called as 'incets' later in the manuscript. How about labeling these as a) and b)? Here, y-axis is not labeled. It is not clear what it means by the intersection for the curve and what it represents biologically. Green or red starts and the values corresponding to it is never explained in the caption.
- Basically figure 2 is an identical tree as what is presented in figure 1. Again, the caption is very difficult to understand. Red and green stars and the values associated with them are never explained. The three values at the node in the tree are never explained.
- Figure 3 has similar problems. More work is needed in presenting the trees. Also, the authors mention in the caption of Figure 3 that the rate is a 'little' slower than that of in Figures 1 and 2, but it is what it means by 'a little', which is a subjective description. 
- The authors mention that the tree topology (based on COI) was unaffected by 18S rRNA. In other words, Figures 1, 2, and 3 are the same trees. If this is true, I am not sure presenting three identical trees is a good practice. Moreover, the authors must explore if the congruence occurred because they actually are congruent or simply the signals in 18S rRNA is too small to override the signals in COI. It would be interesting to explore the tree based on 18S rRNA.
- typo {Abstract} parapheletic -> paraphyletic.
- typo {Figure 2} OUTs -> OTUs.
- substrituions/site/myr and s/s/myr are used interchangeably. It would be a good idea to define for the first time and stick to the abbreviated version.
- Table 1 has a header labeled as 'country', but in fact, 'sampling locality' might be a better description of the content. Also, it might be a better to specify what accession numbers the authors are referring to, GenBank?
- Table 2 caption must be improved. 
- It is unclear how the authors decided to use HKY model for the BEAST analysis.
- The authors mentioned that the 95\% highest posterior density for confidence intervals of ages are not shown in Figure 1--3 to avoid visual complexity. Is it going to be available in supplementary material? This is important information to verify authors' claims regarding the node ages in the manuscript.
- Figure 4 mentions the number of base changes for whole mitochondrial gene, but the two plots are based only on COI and 18S rRNA. It's confusing which is correct. The caption needs improvement.
- In results, the authors refer to Figure 3 as COI + 16S rRNA. It seemed like the authors were working on 18S rRNA. It's confusing which is correct.
- The authors mentioned that the rate has exponentially increased into Holocene based on the incets of Figure 1--3. But no Holocene was labeled anywhere in these figures. Also, the authors stated the curves and rates are similar for the incets in Figure 1,2 and 3, but it is hard to understand how the authors concluded that they are 'similar', which is a very subjective description. 

Is the work clearly and accurately presented and does it cite the current literature?

Partly

If applicable, is the statistical analysis and its interpretation appropriate?

Partly

Are all the source data underlying the results available to ensure full reproducibility?

Partly

Is the study design appropriate and is the work technically sound?

Partly

Are the conclusions drawn adequately supported by the results?

Partly

Are sufficient details of methods and analysis provided to allow replication by others?

Yes

Reviewer Expertise:

Phylogenetics

I confirm that I have read this submission and believe that I have an appropriate level of expertise to state that I do not consider it to be of an acceptable scientific standard, for reasons outlined above.

# Response

**Published:** 2023-07-03

**Authors:** Soichi Osozawa

**Affiliations:** Tohoku University, Japan

copyright

 30 Jun 2023 | for Version 1

 Sungsik Kong, University of Wisconsin-Madison, Madison, Wisconsin, USA 

 format_quote Cite this report

 speaker_notes Responses(0)

 NOT APPROVED

 info_outline

 The main objective of this study is to present a time-calibrated tree of cicada species reconstructed using BEAST v1.X. The trees contain crypic cicada species from Ryukyu islands in addition to the existing data presented in the previous studies. The authors show improved taxonomy of some of the major cicada groups. Moreover the authors suggest an exponential increase of base substitution rate in recent geologic time. While the content of the manuscript is interesting, it requires substantial improvement and is not ready for indexing. The following comments elaborate some places where the improvements can be made.

*Thank you for your review, and we appreciate your attention to our main insight and new finding regarding the exponential increase of base substitution rate in recent geologic time. Although we acknowledge that our experience with BEAST v1. X and the calibration method is limited, we recommend that you become a BEAST v1.X user, as it will provide you with the tools and resources to address your queries effectively. Utilizing BEAST v1.X will enable you to delve deeper into the subject matter and gain a better understanding of the findings we presented.*

 In general, the manuscript is not easy to understand. It is understandable that English may not be the authors' first language, however, an academic manuscript must be written clearly.

*We appreciate your confidence in the writing quality of the present manuscript. Considering that John Wakabayashi is a native English speaker, it is likely that the manuscript is indeed well-written. However, we understand your desire for a thorough review of the English language in the R1 manuscripts.*

 For example, the last sentence of Abstract seems to be not connecting with the previous sentences.

*“We estimated base substitution rate as a function of age, and the result strongly indicates an exponential increase of base substitution rate in recent geologic time.” Is connected to next sentence “The consequent increase in cicada biodiversity, including generation of cryptic species in the Ryukyu Islands and surroundings, may have been driven by the generation and spreading of C4 grasses and coeval Quaternary climate change.”*

 The first sentence of Introduction is excessively long. 

*It is crucial to emphasize the specific type of gene used in the phylogenetic analyses, and our current COI and 18S rRNA sequence data may not be sufficient for our study. Addressing these concerns, we reviewed here the significance of utilizing an appropriate and comprehensive set of genes for the recent trend of phylogenetic analyses for cicadas.*

 In figure 1, is not clear what it means by "maximum" COI sequence.

*“1,534 bp in maximum”, not “maximum COI sequence”. We will revise in R1 manuscript.*

 It is not clear what is the inserted figure. I assume it is the plot on the left side with white background. These are also called as 'inserts' later in the manuscript. How about labeling these as a) and b)? Here, y-axis is not labeled.

*The inset was made by node data shown in Fig. 2. In R1, we will improve the expression. Y axis is Ma, or you questioned no species name?*

 It is not clear what it means by the intersection for the curve and what it represents biologically.

*To draw the approximate curve, better to apply the intersection data, and we applied rate median obtained from Tracer.*

 Green or red starts and the values corresponding to it is never explained in the caption.

*You will find the captions within Fig. 1, but we will enlarge the caption in R1.*

 Basically figure 2 is an identical tree as what is presented in figure 1.

*We acknowledge the feedback provided by Jessica Ware, a reviewer of our dragonfly manuscript, regarding Figure 2 being overly crowded. To address this concern, we have made revisions by dividing the scientific content of Figure 2 into simplified and full version figures, namely Figures 1 and 2, respectively. It is important to note that both figures are identical, as you correctly pointed out.*

*By providing simplified and full versions of the figure, we aim to improve the clarity and readability of the scientific information presented. This division allows readers to quickly grasp the main concepts in the simplified version, while still having access to the more detailed information in the full version if desired.*

*We appreciate the valuable input from Jessica Ware and are grateful for her suggestion to enhance the visual presentation of our manuscript. Thank you for highlighting this issue, and we hope these revisions address the concern effectively.*

 Again, the caption is very difficult to understand. Red and green stars and the values associated with them are never explained. The three values at the node in the tree are never explained.

*You will find the captions within Fig. 2,  but we will enlarge the caption in R1. Probably see also main text.*

 Figure 3 has similar problems. More work is needed in presenting the trees.

*Fig. 3 was presented to compare COI + 18S rRNA tree vs solely COI tree in Figs. 1 and 3.*

*Allow us here too busy figure without simplified figure.*

 Also, the authors mention in the caption of Figure 3 that the rate is a 'little' slower than that of in Figures 1 and 2, but it is what it means by 'a little', which is a subjective description. 

*Rate median 0.0114 in Fig. 3 inset is a little slower than 0.0128 in Figs. 1 and 2, reflecting Fig. 3 was considered slower rated (not sensitive) 18S rRNA data.*

 The authors mention that the tree topology (based on COI) was unaffected by 18S rRNA. In other words, Figures 1, 2, and 3 are the same trees. If this is true, I am not sure presenting three identical trees is a good practice. Moreover, the authors must explore if the congruence occurred because they actually are congruent or simply the signals in 18S rRNA is too small to override the signals in COI. It would be interesting to explore the tree based on 18S rRNA.

*Note that COI tree is for 352 specimens and tips, and COI + 18S rRNA tree is for 1492 specimens and tips, and not strictly identical and congruent.*

*18S rRNA tree is “low resolution” and discordant COI tree. Seealso  “Why was BEAST2 not used”.*

 typo {Abstract} parapheletic -> paraphyletic.

*In R1, we revise.*

 typo {Figure 2} OUTs -> OTUs.

*In R1, we revise.*

 substrituions/site/myr and s/s/myr are used interchangeably. It would be a good idea to define for the first time and stick to the abbreviated version.

*We will delete later appeared “substrituions/site/myr”.*

*Some journal ask to offer abbreviations, but now not a case.*

 Table 1 has a header labeled as 'country', but in fact, 'sampling locality' might be a better description of the content. Also, it might be a better to specify what accession numbers the authors are referring to, GenBank?

*In GenBank/DDJB “country” is requested to use.*

*Also our data with accession numbers are found in GenBank/DDBJ.*

 Table 2 caption must be improved. 

*In R1, we will check English.*

 It is unclear how the authors decided to use HKY model for the BEAST analysis.

*Default setting.*

 The authors mentioned that the 95\% highest posterior density for confidence intervals of ages are not shown in Figure 1--3 to avoid visual complexity. Is it going to be available in supplementary material? This is important information to verify authors' claims regarding the node ages in the manuscript.

*Please consider adding 95% highest posterior density (HPD) bars to represent confidence intervals for ages on each node in Figure 2. The figure becomes further busy. We believe they may not add significant meaning.*

 Figure 4 mentions the number of base changes for whole mitochondrial gene, but the two plots are based only on COI and 18S rRNA. It's confusing which is correct. The caption needs improvement.

*Vertical axis is COI numbers of base changes and 18S rRNA numbers of base changes, respectively in left sided COI and right sided 18S rRNA figures.*

 In results, the authors refer to Figure 3 as COI + 16S rRNA. It seemed like the authors were working on 18S rRNA. It's confusing which is correct.

*We found four mistakes of 16S rRNA in page 11 including Osozawa et al. (2016), and we collect as 18S rRNA and Osozawa et al. (2017a) in R1.*

 The authors mentioned that the rate has exponentially increased into Holocene based on the incets of Figure 1--3. But no Holocene was labeled anywhere in these figures. Also, the authors stated the curves and rates are similar for the incets in Figure 1,2 and 3, but it is hard to understand how the authors concluded that they are 'similar', which is a very subjective description. 

*The Holocene is the most recent epoch of the Quaternary period, following the boundary at 0.0117 Ma. We acknowledge the difficulty in accurately labeling the time point at 0.0117 Ma in Figures 1 to 3.*

*Compare formula in the same one in Fig. 1 and 2, and the distinct one in Fig. 3.*

*We were hoping your constructive review about increasing rate, factor, and affect.*

 Is the work clearly and accurately presented and does it cite the current literature?

 Partly

 Is the study design appropriate and is the work technically sound?

 Partly

 Are sufficient details of methods and analysis provided to allow replication by others?

 Yes

 If applicable, is the statistical analysis and its interpretation appropriate?

 Partly

 Are all the source data underlying the results available to ensure full reproducibility?

 Partly

 Are the conclusions drawn adequately supported by the results?

 Partly

 Competing Interests

 No competing interests were disclosed.

 Reviewer Expertise

 Phylogenetics

 I confirm that I have read this submission and believe that I have an appropriate level of expertise to state that I do not consider it to be of an acceptable scientific standard, for reasons outlined above.
